# Author Correction: Allostery can convert binding free energies into concerted domain motions in enzymes

**DOI:** 10.1038/s41467-024-55735-4

**Published:** 2025-01-15

**Authors:** Nicole Stéphanie Galenkamp, Sarah Zernia, Yulan B. Van Oppen, Marco van den Noort, Andreas Milias-Argeitis, Giovanni Maglia

**Affiliations:** 1 Chemical Biology I, Groningen Biomolecular Sciences & Biotechnology Institute, University of Groningen, 9747 AG Groningen, The Netherlands; 2 Molecular Systems Biology, Groningen Biomolecular Sciences & Biotechnology Institute, University of Groningen, 9747 AG Groningen, The Netherlands

**Keywords:** Single-molecule biophysics, Kinases, Enzyme mechanisms

Correction to: *Nature Communications* 10.1038/s41467-024-54421-9, published online 22 November 2024

In this article the author’s name Andreas Milias-Argeitis was incorrectly written as Andreas Milias Argeitis. The original article has been corrected.

