# Supplementary material for: Allostery can convert binding free energies into concerted domain motions in enzymes
Source: Nat Commun. 2024 Nov 22;15:10109. doi: 10.1038/s41467-024-54421-9 (PMC11582565; doi:10.1038/s41467-024-54421-9)
Supplement: Supplementary file 1 — Supplementary Information [file 41467_2024_54421_MOESM1_ESM.pdf]

SUPPLEMENTARY INFORMATION

Allostery can convert binding free energies into concerted domain motions in enzymes

Nicole Stéphanie Galenkamp*[1], Sarah Zernia*[1], Yulan B. Van Oppen[2], Marco van den Noort[1], Andreas Milias Argeitis[2] & Giovanni Maglia[#1]

[1]Chemical biology, Groningen Biomolecular Sciences & Biotechnology Institute, University of Groningen, 9747 AG Groningen, The Netherlands
[2]Molecular Systems Biology, Groningen Biomolecular Sciences & Biotechnology Institute, University of Groningen, 9747 AG Groningen, The Netherlands

*Equal contribution
#Corresponding author: giovanni.maglia@rug.nl

Supplementary discussion

Activity of AK_2K

The activity of AK variants was tested as described in details earlier[1–3]. In short, a coupled assay was performed whereby the backward reaction (ADP+ADP+Mg^{2+}→ ATP+AMP+Mg^{2+}) was measured by following the reduction of $NADP^+$ to NADPH at 340 nm. Hereby the formed ATP by the adenylate kinase, together with glucose was converted to ADP and glucose-6-phosphate. After, the glucose-6-phosphate was reacted to 6-phospho-glucono-D-glucono-1,5-lactone by glucose-6-phosophate dehydrogenase, while using $NADP^+$ as a factor, with NAPDH, the readout component, as product (Supplementary Figure 1).

Adenylate kinase occupies three different binding positions in the nanopore.

AK showed three types of characteristic signals named Type-a, -b and -c blockades. At low potential (-50 mV), mostly type-b blockades were observed (84.0 ± 14.3 %), while increasing the applied potential, the other two blockades appeared. At -90 mV Type-a and Type-b blockades were almost equally represented (35.4 ± 7.1% and 46.7 ± 15.7 % of the time, respectively), and low-noise signals (I_{RES} of 46.0 ± 0.1 % and 58.5 ± 0.3 %, respectively, Supplementary Figure 2, 400 mM KCl, 15 mM Tris, 2 mM $MgCl_2$, pH 7.5). Type-c blockades represented only about 18% of the total and showed higher current noise (Supplementary Figure 2). The dwell time of Type-c and Type-b blockades showed a maximum at ~-70 mV, suggesting that the protein translocated above that threshold potential. The dwell time of Type-a blockades increased with the potential (Supplementary Figure 2), indicating that this protein group did not translocate across the nanopore. These blockades can be due to a protein impurity or the entry of AK inside the nanopore with a different position/orientation (Supplementary Figure 2). The addition of P^1,P^5-Di-(adenosine-5')-pentaphosphate (Ap5A), an inhibitor of AK, to either the *trans* or *cis* side of the nanopore changed Type-a and Type-b blockades (Supplementary Figure 4), suggesting that these blockades represent AK. Type-a blockades fluctuated between five interconverting current levels: M, M1, M2, M3 and M4 (I_{RES_M1} = 46.3 ± 0.2 %, I_{RES_M2} = 48.6 ± 0.2 %, I_{RES_M3} = 51.0 ± 0.1 %, I_{RES_M4} = 53.6 ± 0.1 %, respectively, Figure 2B, Supplementary Figure 5, Supplementary Table 2), while Type-b blockade showed one additional current level (I_{RES} = 56.6 ± 0.2). Previously, thrombin and haemoglobin have shown to induce two current blockades by interacting with different binding sites inside the ClyA and PlyAB nanopore, respectively[4,5]. The current blockades showed a voltage dependency with the binding to shallower sites being more prominent at lower applied potentials. In another case, it was shown that SBD2 can enter in two different orientations within the ClyA nanopore, which induce two separate current levels[6]. Given that Type-a and -b blockades reacted to the inhibitor, we think the most likely explanation is that they represent AK entering inside the nanopore with two different orientations. Type-c blockades remained unchanged by Ap5A suggesting they represented misfolded proteins or impurities from the protein preparation.

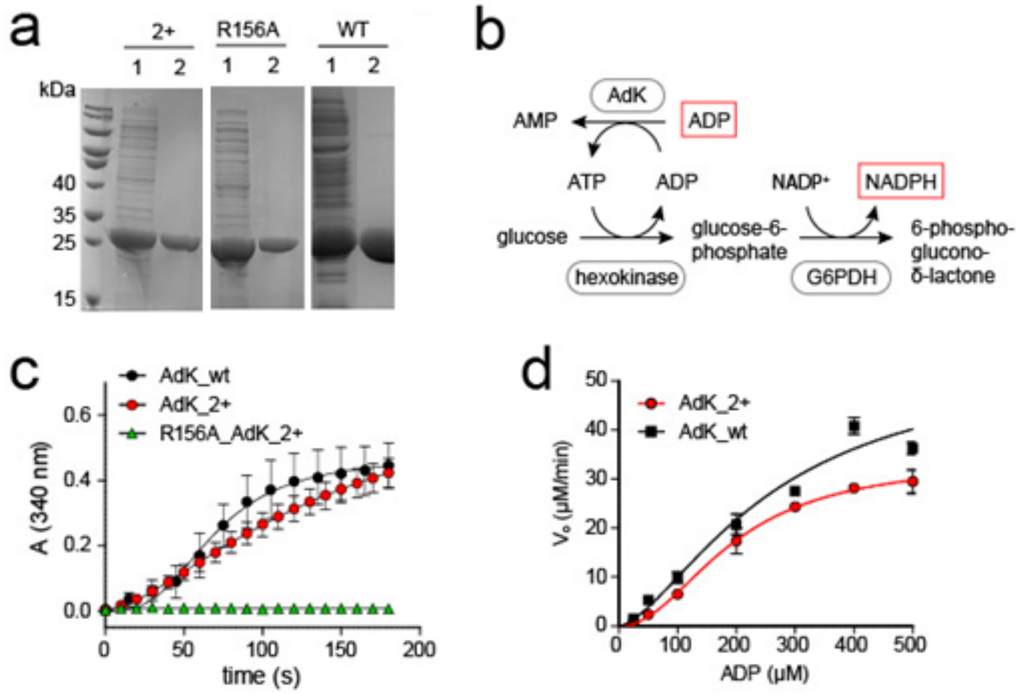

Supplementary Figure 1. Protein production and bulk activity. (a) SDS-PAGE of AK variants before (1) and after (2) affinity-based purification on Strep-tactin sepharose: 2+= AK_2+; R156A= R156A_AK_2+; WT= wild-type AK. Standard: Page Ruler prestained, staining: Coomassie. **(b)** Reaction scheme of bulk activity assay. **(c)** Time-dependent bulk activity assay of AK variants in 400 mM KCl, 15 mM Tris, 2 mM $MgCl_2$, pH 7.5 with ADP as substrate. Data points represent the mean ± SEM (n = 3). **(d)** Concentration-dependent bulk activity assay of AK_wt and AK_2+. Initial velocity of time-dependent measurements was fitted to a hill equation.

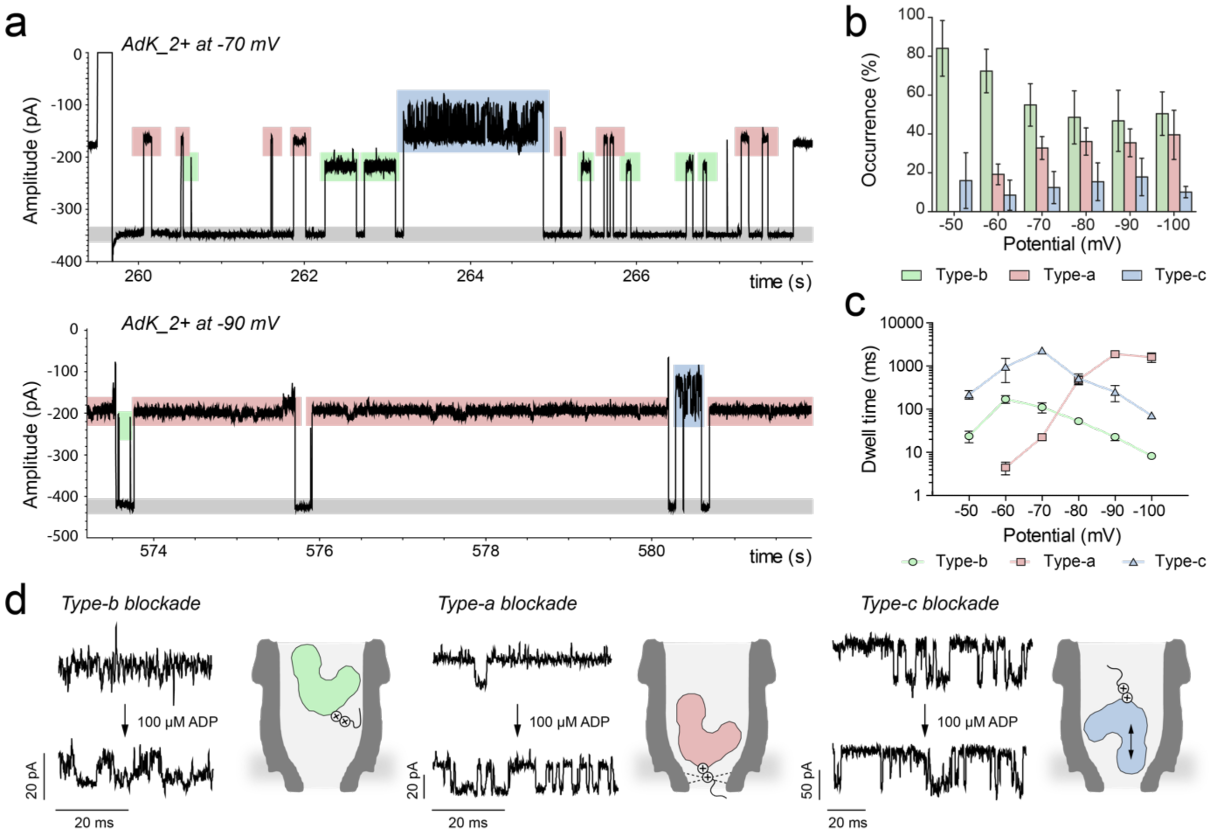

Supplementary Figure 2. AK_2+ signal in the ClyA-AS nanopore. (a) Example traces of AK_2+ in ClyA-AS at two different potentials. The open pore current (I_O) is labelled in grey. Three types of protein blockades (I_B) could be identified, named Type-a (red), Type-b (green), and Type-c (blue). The experiments were performed in 400 mM KCl, 15 mM Tris, 2 mM $MgCl_2$, pH 7.5 with a negative bias (*trans*), 10 kHz sampling rate and 2 kHz low-pass Bessel filter. For figure preparation, traces were additionally filtered with a 500 Hz Gaussian filter. **(b)** Appearance of the three different blockade types. Individual blockades were counted and compared to the sum of all blockades. **(c)** Mean dwell times of the three different blockade types in dependency of the applied potential. **(b+c)** Data points represent the mean ± SEM (n = 3). **(d)** Ligand response of individual blockade types. Protein was added from *cis*, 100 µM ADP from *trans*, -90 mV was applied (*trans*). Schemes show the interpretation of the different blockade types of AK_2+ (green, red or blue, respectively) in the ClyA nanopore (grey).

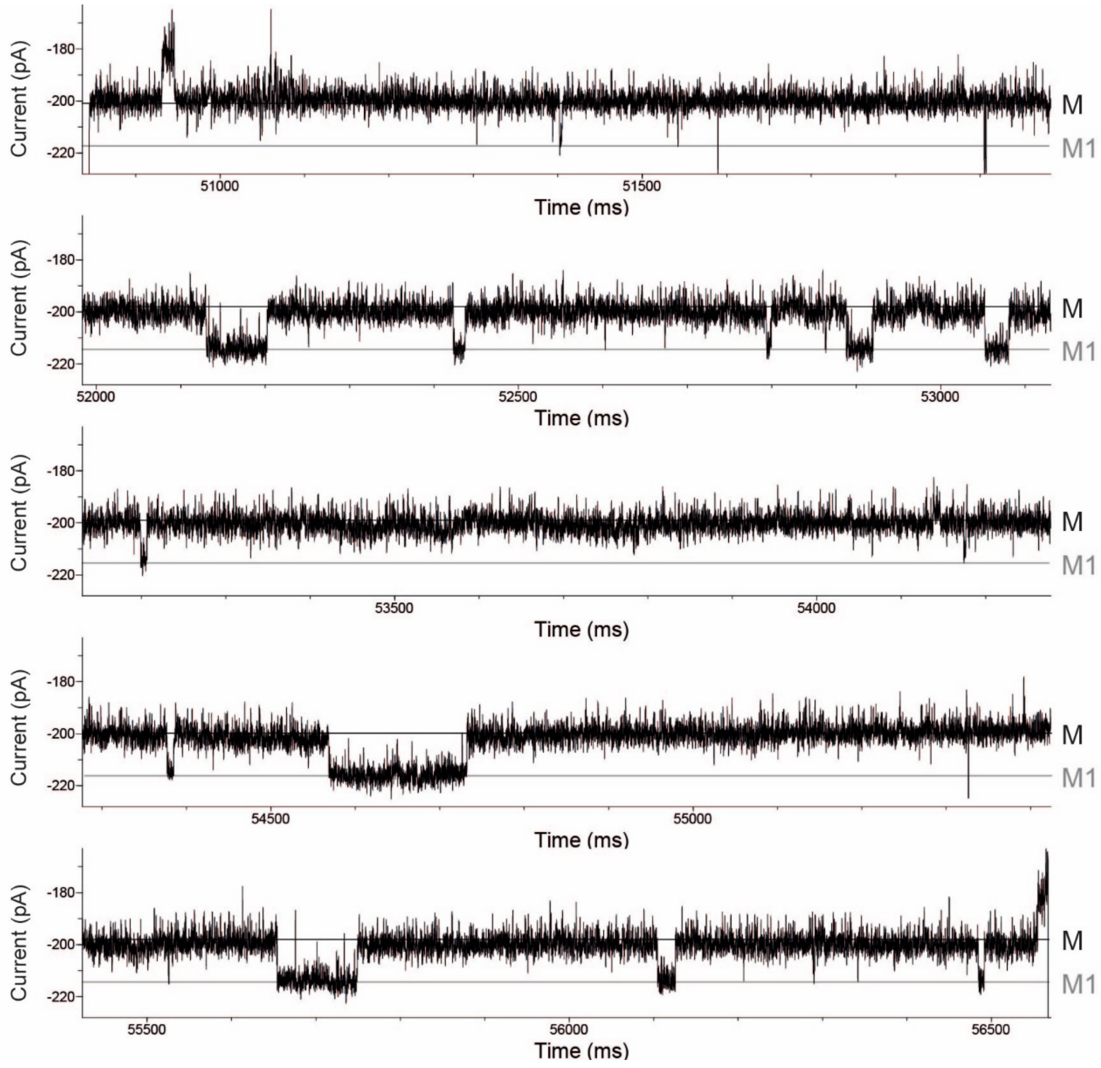

Supplementary Figure 3. Continuous recording of AK_2+ conformational changes of the *apo*-enzyme. M and M1 represent the open conformation (black and grey line). The measurements were performed in 400 mM KCl, 15 mM Tris, 2 mM $MgCl_2$, pH 7.5 at room temperature (22°C) applying -90 mV (*trans*) and sampling at 50 kHz with a 10 kHz Bessel-low filter, additionally digitally filtered with a 2 kHz Gaussian low-pass filter.

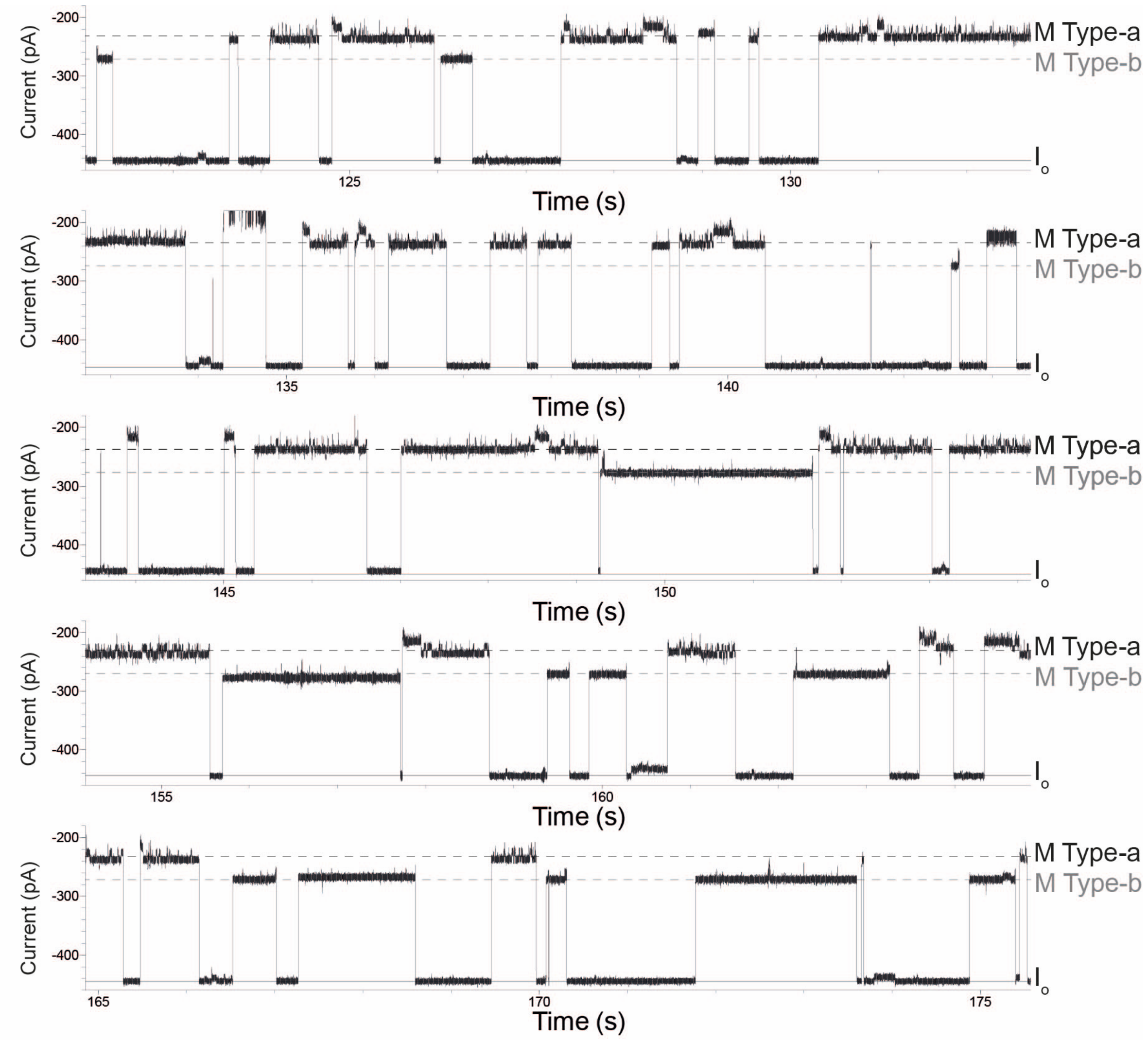

Supplementary Figure 4. AK_2+ current blockades in the ClyA-AS nanopore upon binding of Ap5A. Current trace showing the capture of different AK_2+ types inside ClyA-AS at -90 mV applied potential after addition of 10 µM Ap5A to the *cis* compartment. Two types of AK protein blockades are observed based on residual current. The black dashed line indicates Type-a blockades and the grey dashed line indicates Type-b blockades. The measurements were performed in 400 mM KCl, 15 mM Tris, 2 mM $MgCl_2$, pH 7.5 at room temperature (22°C) applying -90 mV (*trans*) and sampling at 50 kHz with a 10 kHz Bessel-low filter, additionally digitally filtered with a 2 kHz Gaussian low-pass filter.

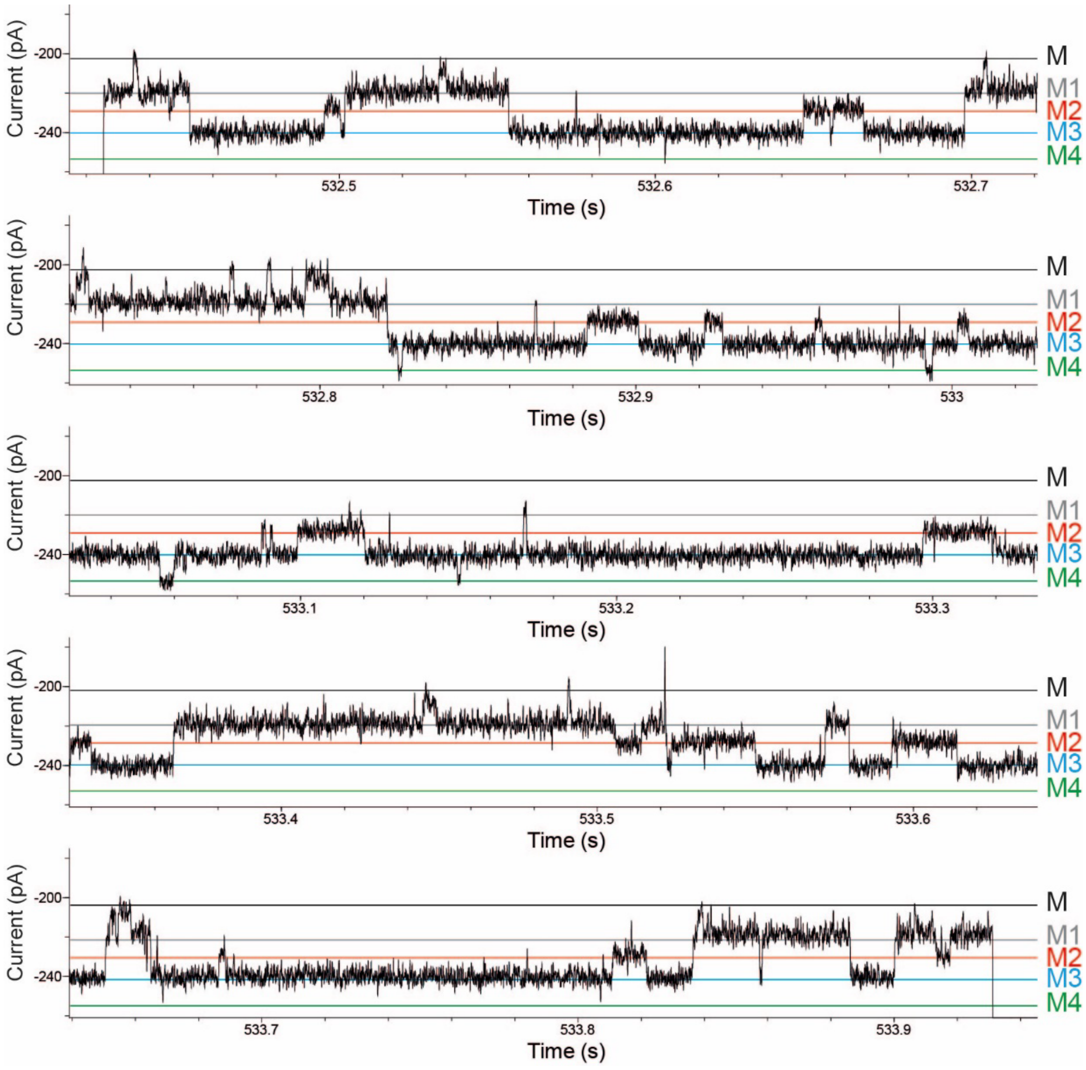

Supplementary Figure 5. Continuous recording of AK_2+ conformational changes during the binding of Ap5A. Ap5A (10 µM) was added to the *trans* chamber. M and M1 represent the open conformation (black and grey line), M2 the protein with the LID domain closed (red line), M3 with both the LID and NMP domain closed (blue line) and M4 with completely closed enzyme (green line). The measurements were performed in 400 mM KCl, 15 mM Tris, 2 mM $MgCl_2$, pH 7.5 at room temperature (22°C) applying -90 mV (*trans*) and sampling at 50 kHz with a 10 kHz Bessel-low filter, additionally digitally filtered with a 2 kHz Gaussian low-pass filter.

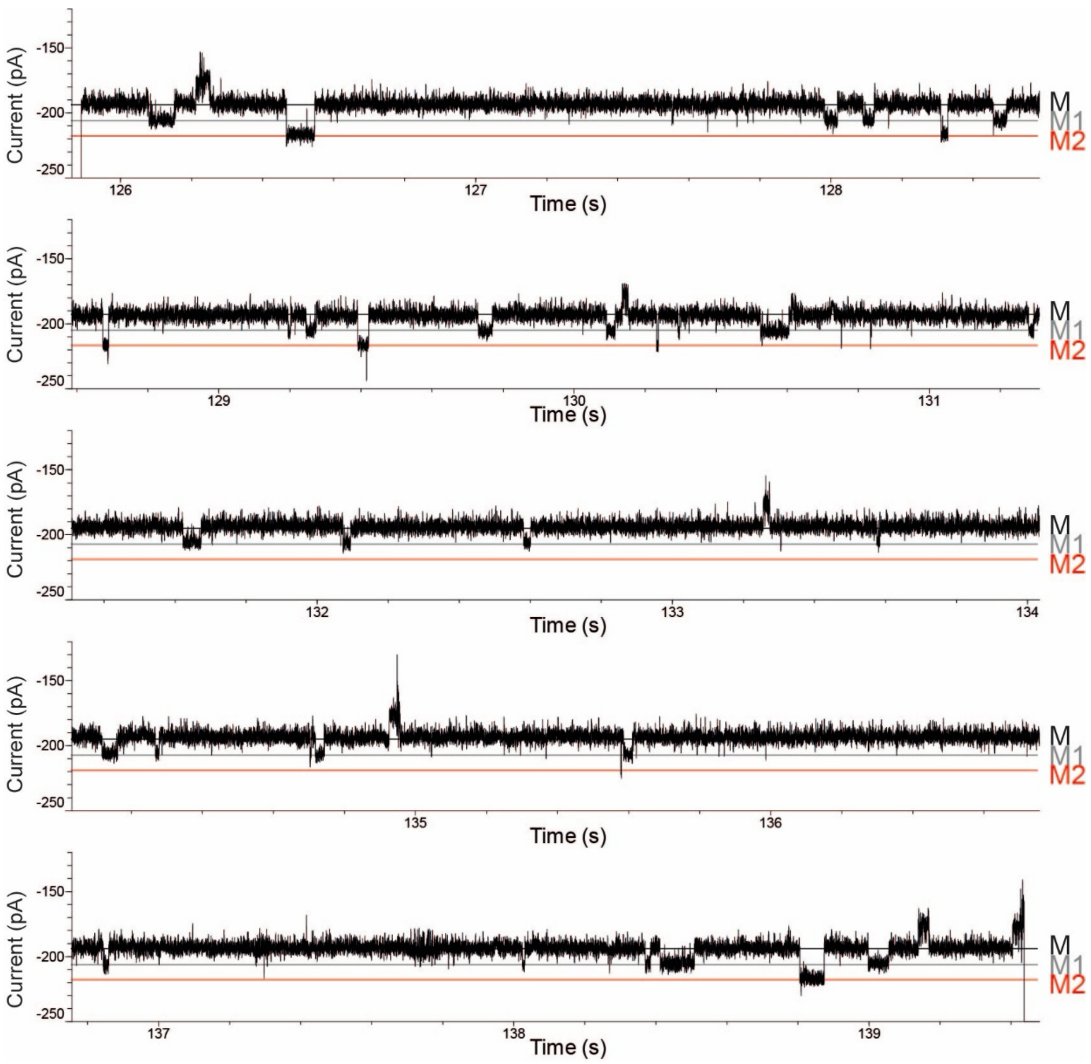

Supplementary Figure 6. Continuous recording of AK_2+ conformational changes during the binding of AMP. AMP (1 mM) was added to the *trans* chamber. M and M1 represent the open conformation (black and grey line) and M2 the protein with the LID domain closed (red line). The measurements were performed in 400 mM KCl, 15 mM Tris, 2 mM $MgCl_2$, pH 7.5 at room temperature (22°C) applying -90 mV (*trans*) and sampling at 50 kHz with a 10 kHz Bessel-low filter, additionally digitally filtered with a 2 kHz Gaussian low-pass filter.

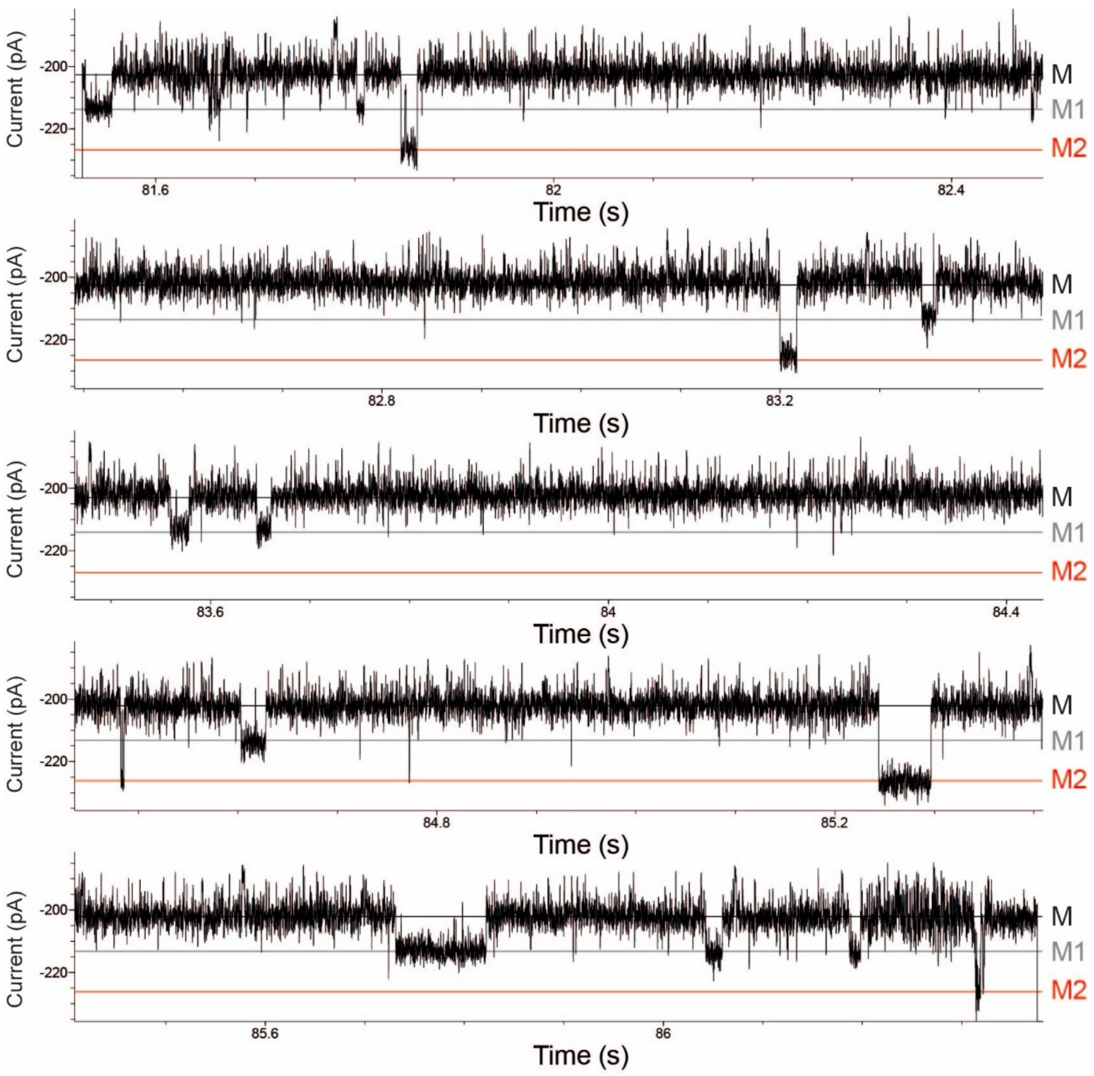

Supplementary Figure 7. Continuous recording of AK_2+ conformational changes during the binding of AMP without Mg^{2+}. AMP (1 mM) was added to the *trans* chamber. M and M1 represent the open conformation (black and grey line) and M2 the protein with the LID domain closed (red line). The measurements were performed in 400 mM KCl, 15 mM Tris, 100 μM EDTA, pH 7.5 at room temperature (22°C) applying -90 mV (*trans*) and sampling at 50 kHz with a 10 kHz Bessel-low filter, additionally digitally filtered with a 2 kHz Gaussian low-pass filter.

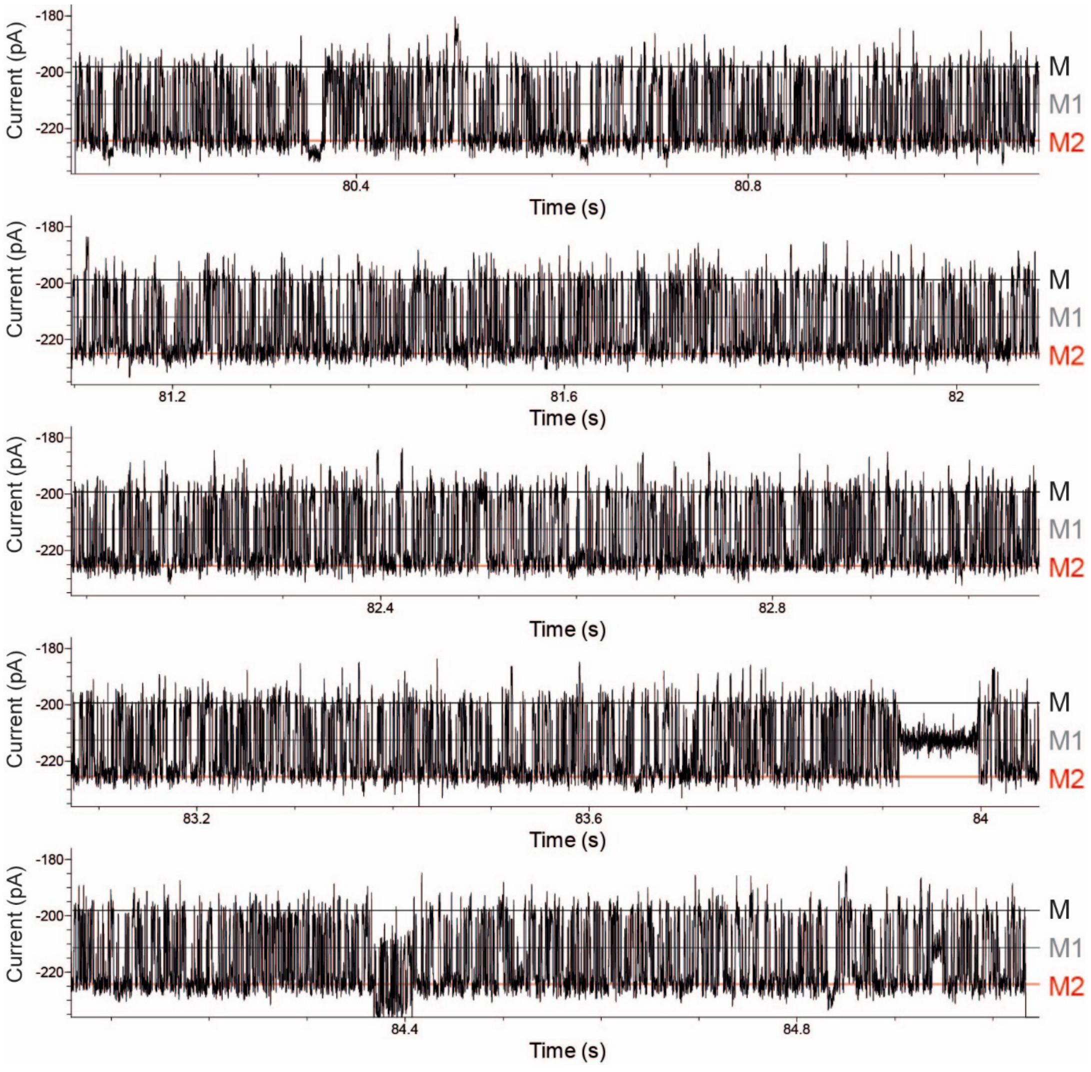

Supplementary Figure 8. Continuous recording of AK_2+ conformational changes during the binding of ATP. ATP (1 mM) was added to the *trans* chamber. M and M1 represent the open conformation (black and grey line) and M2 the protein with the LID domain closed (red line). The measurements were performed in 400 mM KCl, 15 mM Tris, 2 mM $MgCl_2$, pH 7.5 at room temperature (22°C) applying -90 mV (*trans*) and sampling at 50 kHz with a 10 kHz Bessel-low filter, additionally digitally filtered with a 2 kHz Gaussian low-pass filter.

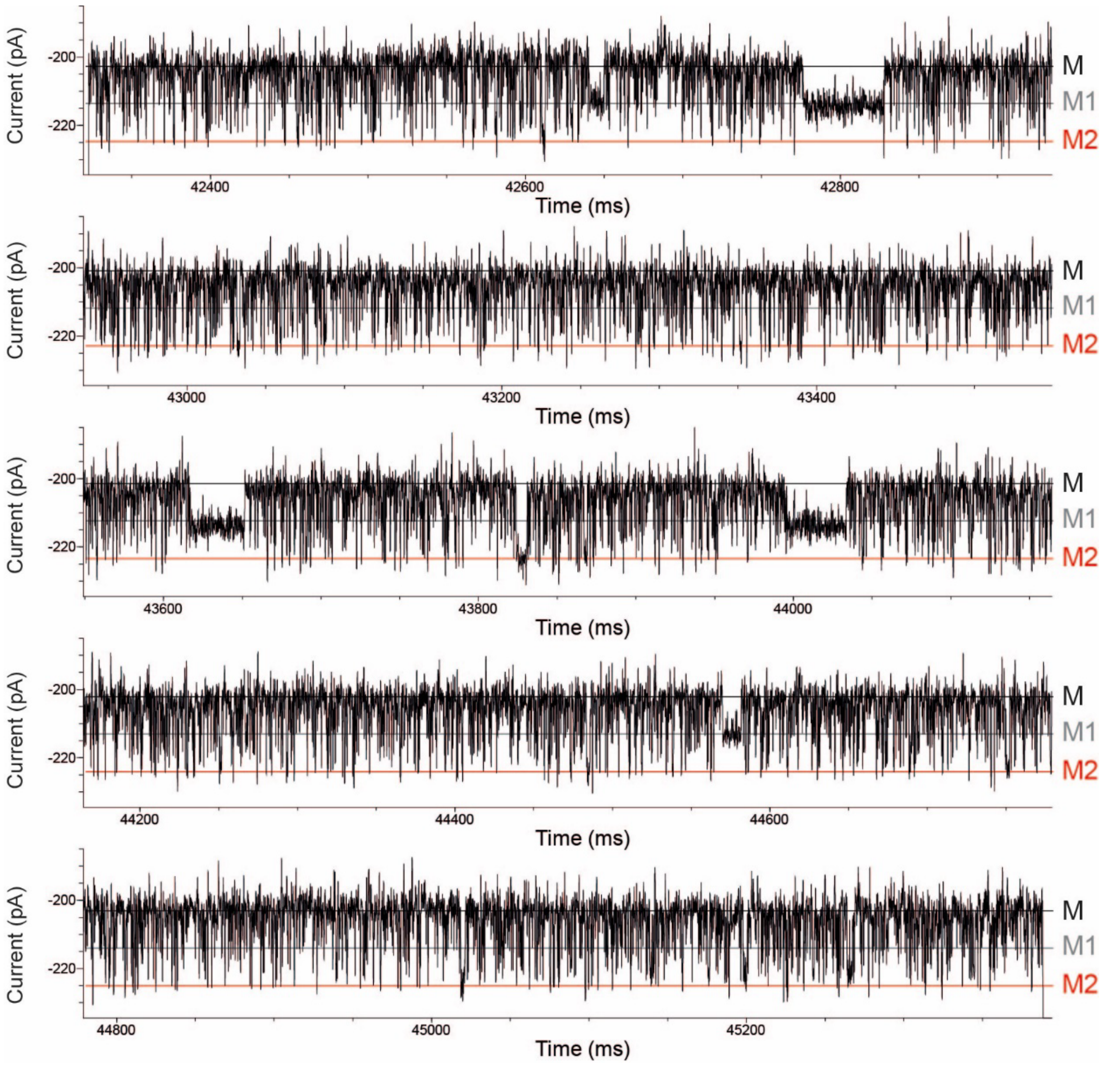

Supplementary Figure 9. Continuous recording of AK_2+ conformational changes during the binding of ATP without Mg^{2+}. ATP (1 mM) was added to the *trans* chamber. M and M1 represent the open conformation (black and grey line) and M2 the protein with the LID domain closed (red line). The measurements were performed in 400 mM KCl, 15 mM Tris, 100 µM EDTA, pH 7.5 at room temperature (22°C) applying -90 mV (*trans*) and sampling at 50 kHz with a 10 kHz Bessel-low filter, additionally digitally filtered with a 2 kHz Gaussian low-pass filter.

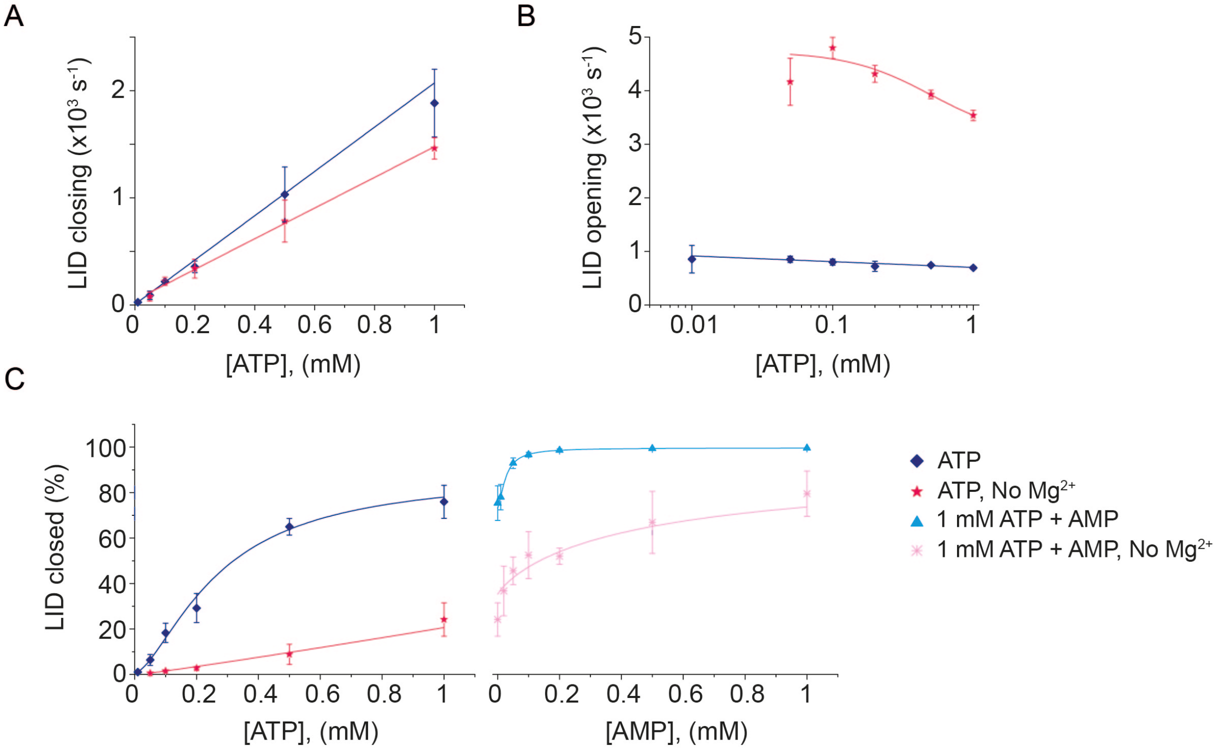

Supplementary Figure 10. Kinetics of ATP binding without Mg^{2+}. (A) Frequency of LID closing - measured as the inverse of dwell times – at increasing ATP concentrations. The lines indicate a linear fit. **(B)** LID opening frequency at increasing ATP concentrations. The lines are fitting the data to a Hill equation. **(C)** Percentage of the closed LID domain at increasing ligand concentrations. The lines indicate fitting to a Hill equation. The measurements were performed in 400 mM KCl, 15 mM Tris, 2 mM $MgCl_2$ (except when omitted), pH 7.5 at room temperature (22°C) applying -90 mV (*trans*) and sampling at 50 kHz with a 10 kHz Bessel-low filter, additionally digitally filtered with a 2 kHz Gaussian low-pass filter. Ligands were added to the *trans* chamber and the enzyme to the *cis* chamber. Error bars in all graphs represents the standard deviation of the mean between independent experiments (N = 3).

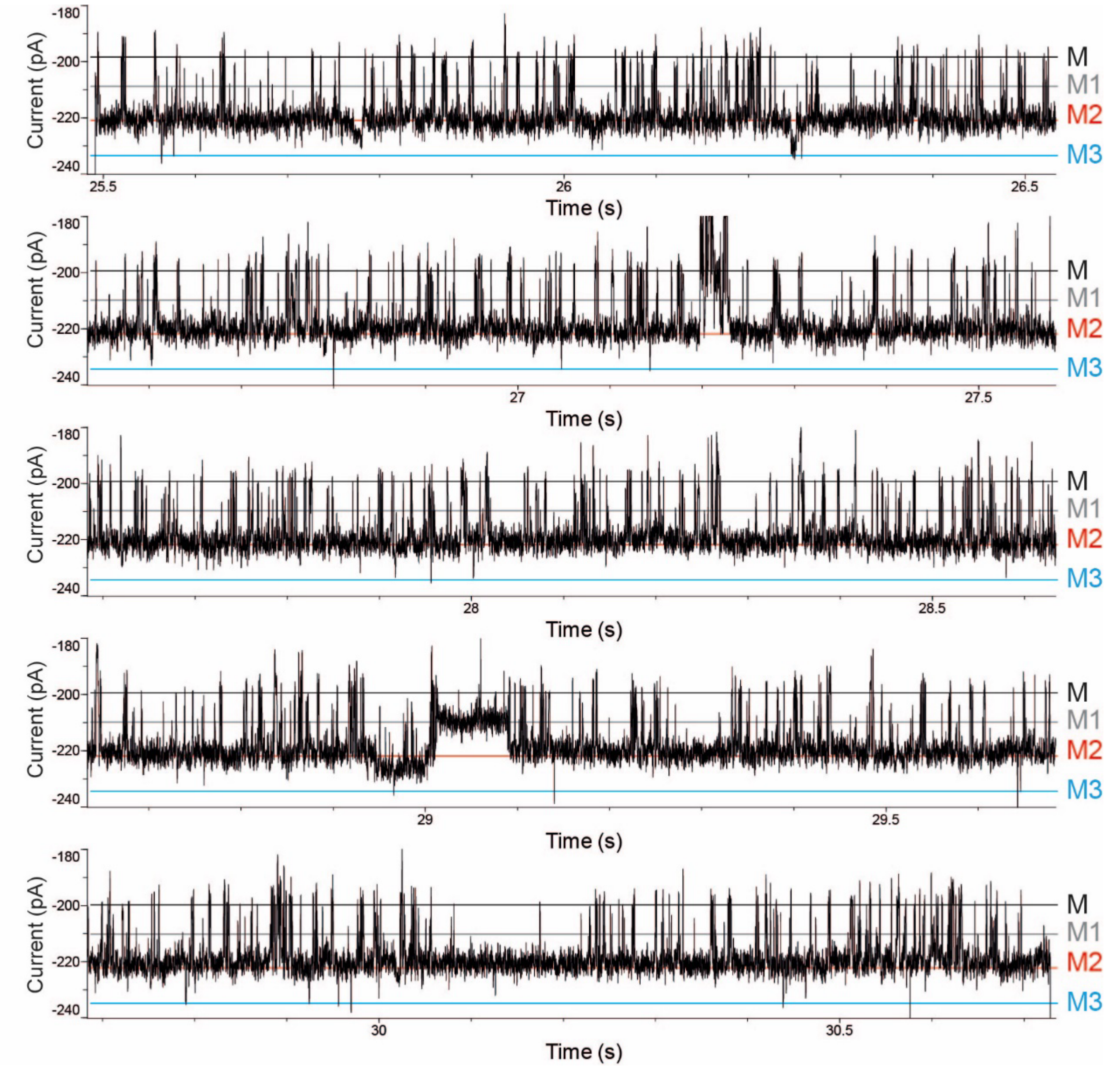

Supplementary Figure 11. Continuous recording of AK_2+ conformational changes during the binding of ATP and AMP. ATP (1 mM) and AMP (100 µM) were added to the *trans* chamber. M and M1 represent the open conformation (black and grey line), M2 the protein with the LID domain closed (red line) and M3 with both the LID and NMP domain closed (blue line). The measurements were performed in 400 mM KCl, 15 mM Tris, 2 mM $MgCl_2$, pH 7.5 at room temperature (22°C) applying -90 mV (*trans*) and sampling at 50 kHz with a 10 kHz Bessel-low filter, additionally digitally filtered with a 2 kHz Gaussian low-pass filter.

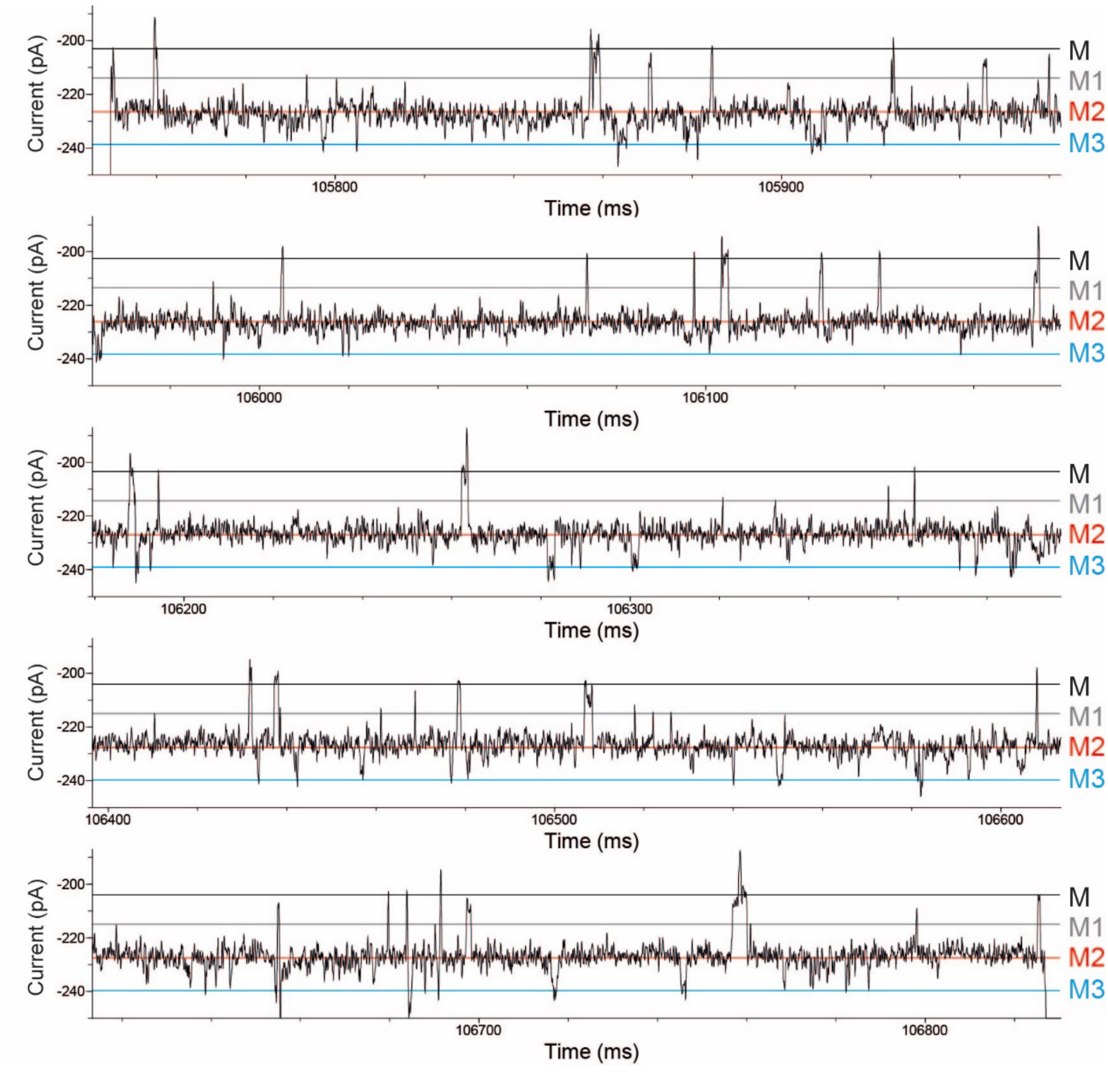

Supplementary Figure 12. Continuous recording of AK_2+ conformational changes during the binding of ATP and AMP. ATP (1 mM) and AMP (1 mM) were added to the *trans* chamber. M and M1 represent the open conformation (black and grey line), M2 the protein with the LID domain closed (red line) and M3 with both the LID and NMP domain closed (blue line). The measurements were performed in 400 mM KCl, 15 mM Tris, 2 mM $MgCl_2$, pH 7.5 at room temperature (22°C) applying -90 mV (*trans*) and sampling at 50 kHz with a 10 kHz Bessel-low filter, additionally digitally filtered with a 2 kHz Gaussian low-pass filter.

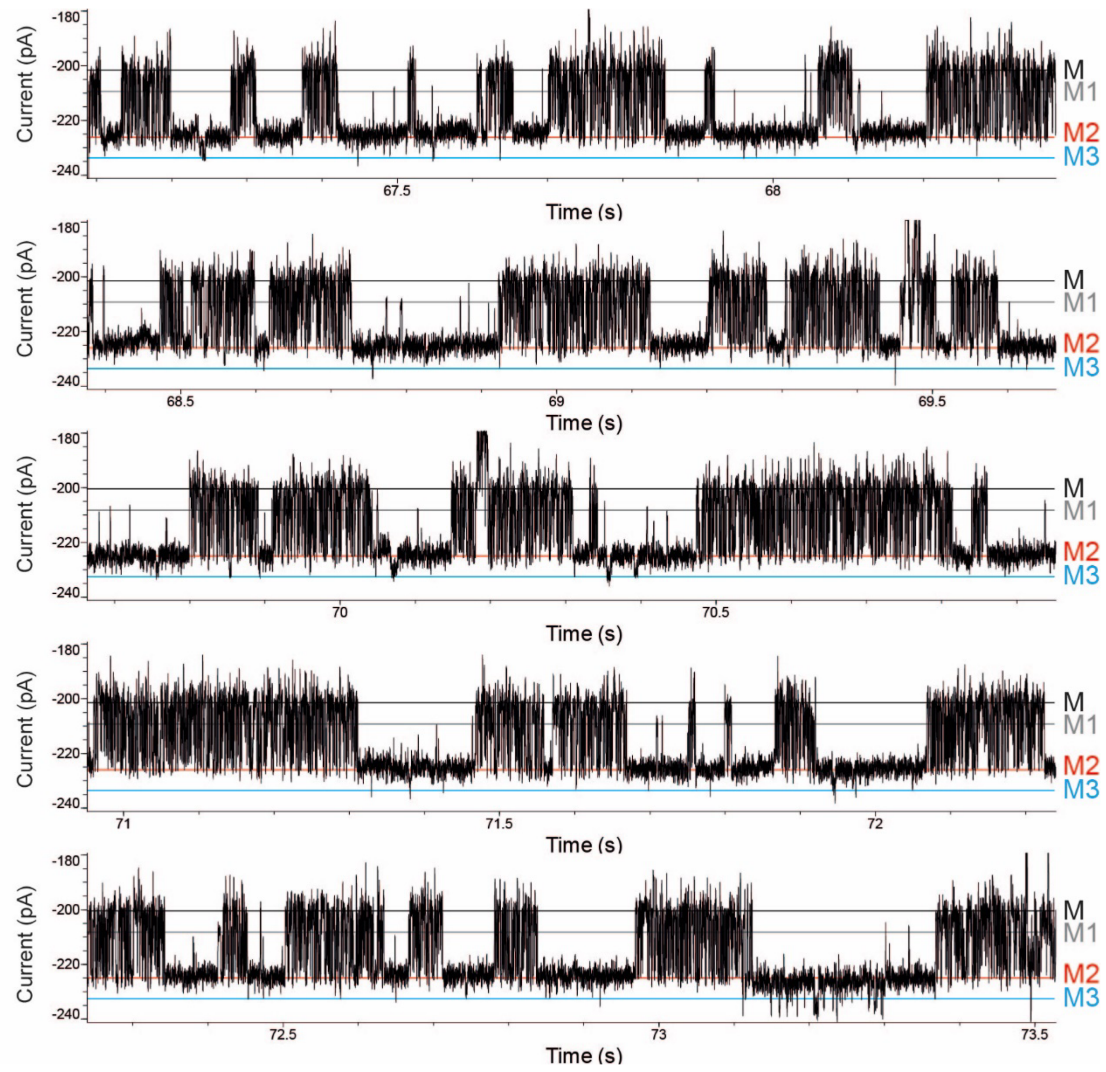

Supplementary Figure 13. Continuous recording of AK_2+ conformational changes during the binding of ATP and AMP without Mg^{2+}. ATP (1 mM) and AMP (1 mM) was added to the *trans* chamber. M and M1 represent the open conformation (black and grey line), M2 the protein with the LID domain closed (red line) and M3 with both the LID and NMP domain closed (blue line). The measurements were performed in 400 mM KCl, 15 mM Tris, 100 µM EDTA, pH 7.5 at room temperature (22°C) applying -90 mV (*trans*) and sampling at 50 kHz with a 10 kHz Bessel-low filter, additionally digitally filtered with a 2 kHz Gaussian low-pass filter.

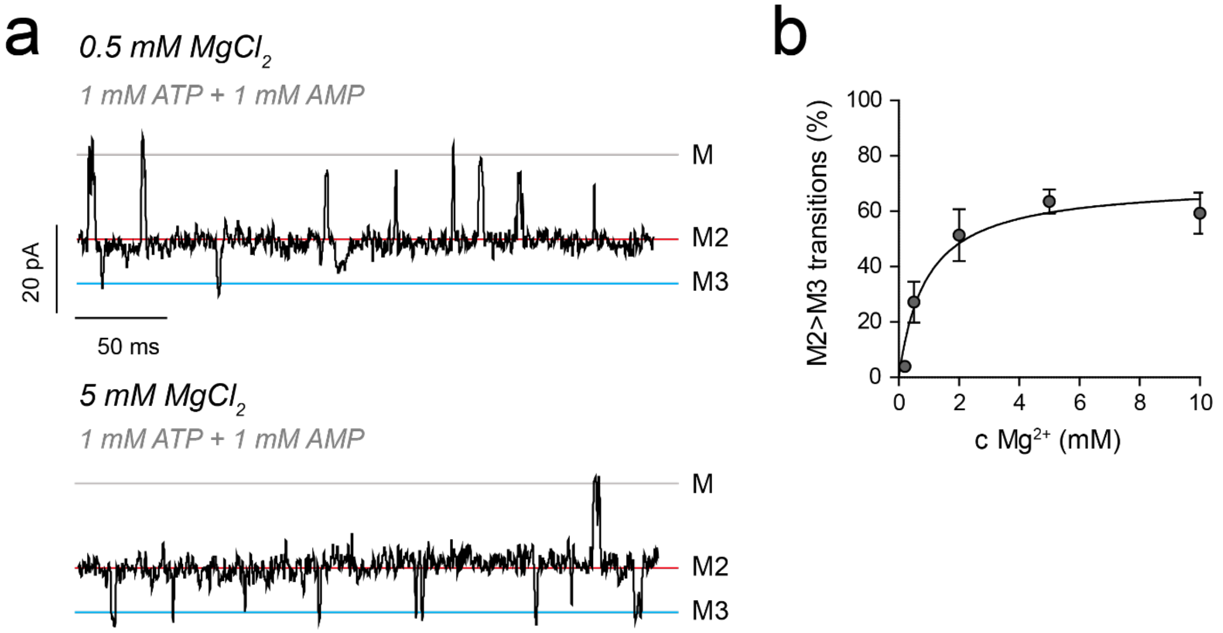

Supplementary Figure 14. Mg^{2+}-dependency of AK_2+ binding behavior. (a) Example traces of AK_2+ (*cis*) with 1 mM ATP + 1 mM AMP (*trans*) at different Mg^{2+} concentrations (*cis* and *trans*). Measurements were performed in 400 mM KCl, 15 mM Tris, 2 mM $MgCl_2$, pH 7.5 with a -90 mV bias (*trans*), 50 kHz sampling rate and 10 kHz low-pass Bessel filtering. Traces were additionally digitally filtered with a 1 kHz Gaussian filter. **(b)** Amount of NMP closing events at 1 mM ATP + 1 mM AMP at increasing Mg^{2+} concentrations determined from counting M3 events in relation to the sum of all events (M, M2 and M3). Percentage was fitted to a hill equation. Data points represent the mean ± SEM (n ≥ 3).

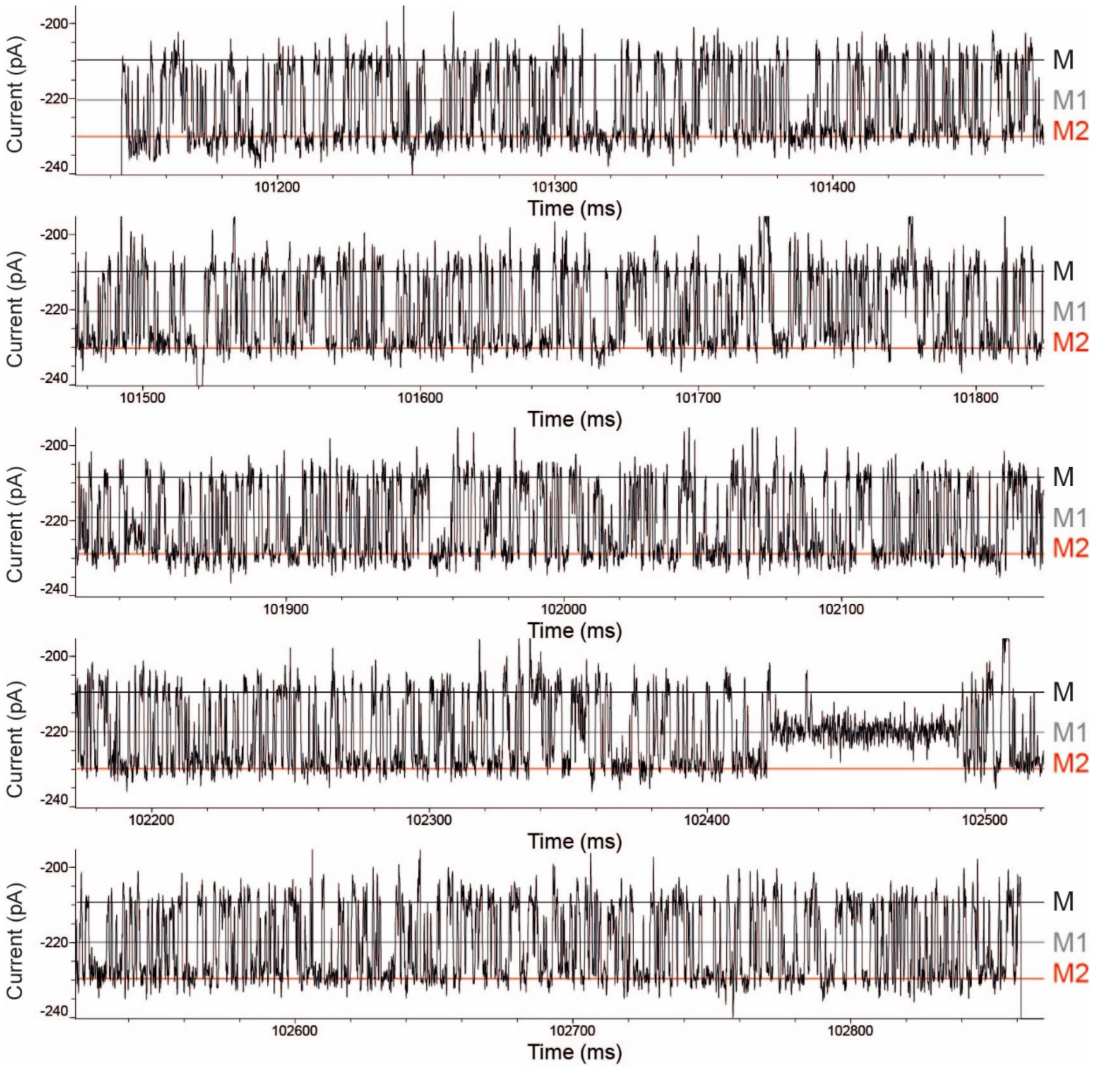

Supplementary Figure 15. Continuous recording of R156A_AK_2+ conformational changes during the binding of ATP. ATP (1 mM) was added to the *trans* chamber. M and M1 represent the open conformation (black and grey line) and M2 the protein with the LID domain closed (red line). The measurements were performed in 400 mM KCl, 15 mM Tris, 2 mM $MgCl_2$, pH 7.5 at room temperature (22°C) applying -90 mV (*trans*) and sampling at 50 kHz with a 10 kHz Bessel-low filter, additionally digitally filtered with a 2 kHz Gaussian low-pass filter.

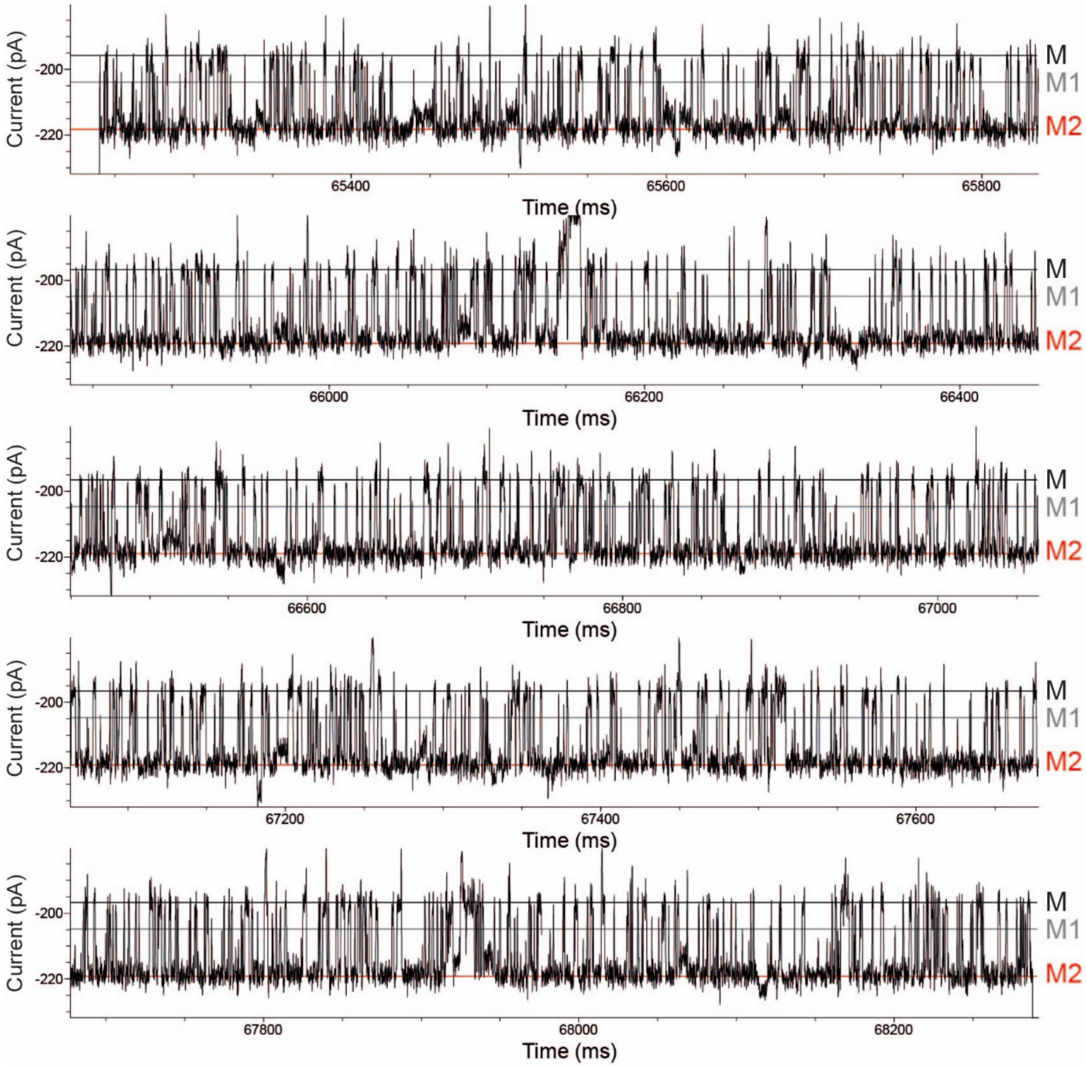

Supplementary Figure 16. Continuous recording of R156A_AK_2+ conformational changes during the binding of ATP and AMP. ATP (1 mM) and AMP (1 mM) were added to the *trans* chamber. M and M1 represent the open conformation (black and grey line) and M2 the protein with the LID domain closed (red line). The measurements were performed in 400 mM KCl, 15 mM Tris, 2 mM $MgCl_2$, pH 7.5 at room temperature (22°C) applying -90 mV (*trans*) and sampling at 50 kHz with a 10 kHz Bessel-low filter, additionally digitally filtered with a 2 kHz Gaussian low-pass filter.

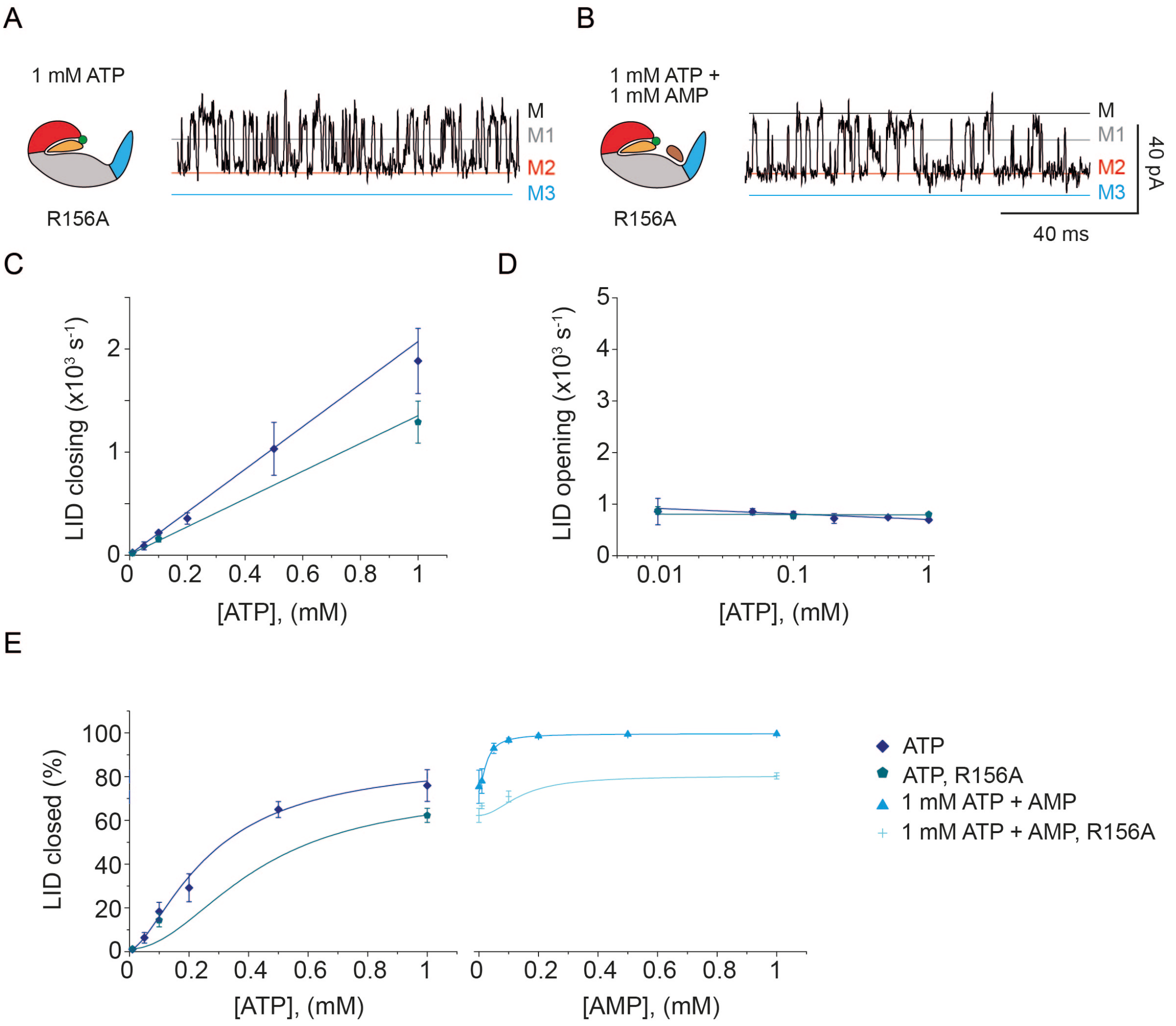

Supplementary Figure 17. R156A_AK_2+ current blockades in the ClyA-AS nanopore upon binding of ATP and AMP. **(A-B)** Typical blockades of R156A_AK_2+ in the presence of 1 mM ATP (**A**), and 1 mM ATP and 1 mM AMP (**B**). **(C)** Frequency of LID closing - measured as the inverse of dwell times – at increasing ATP concentrations. The line indicates a linear fit. **(D)** LID opening frequency at increasing ATP concentrations. The lines are fitting the data to a Hill equation. **(E)** Percentage of the closed LID domain at increasing ligand concentrations. The lines indicate fitting to a Hill function. The measurements were performed in 400 mM KCl, 15 mM Tris, 2 mM $MgCl_2$, pH 7.5 at room temperature (22°C) applying -90 mV (*trans*) and sampling at 50 kHz with a 10 kHz Bessel-low filter, additionally digitally filtered with a 2 kHz Gaussian low-pass filter. Ligands were added to the *trans* chamber and the enzyme to the *cis* chamber. Error bars in all graphs represents the standard deviation of the mean between independent experiments (N = 3).

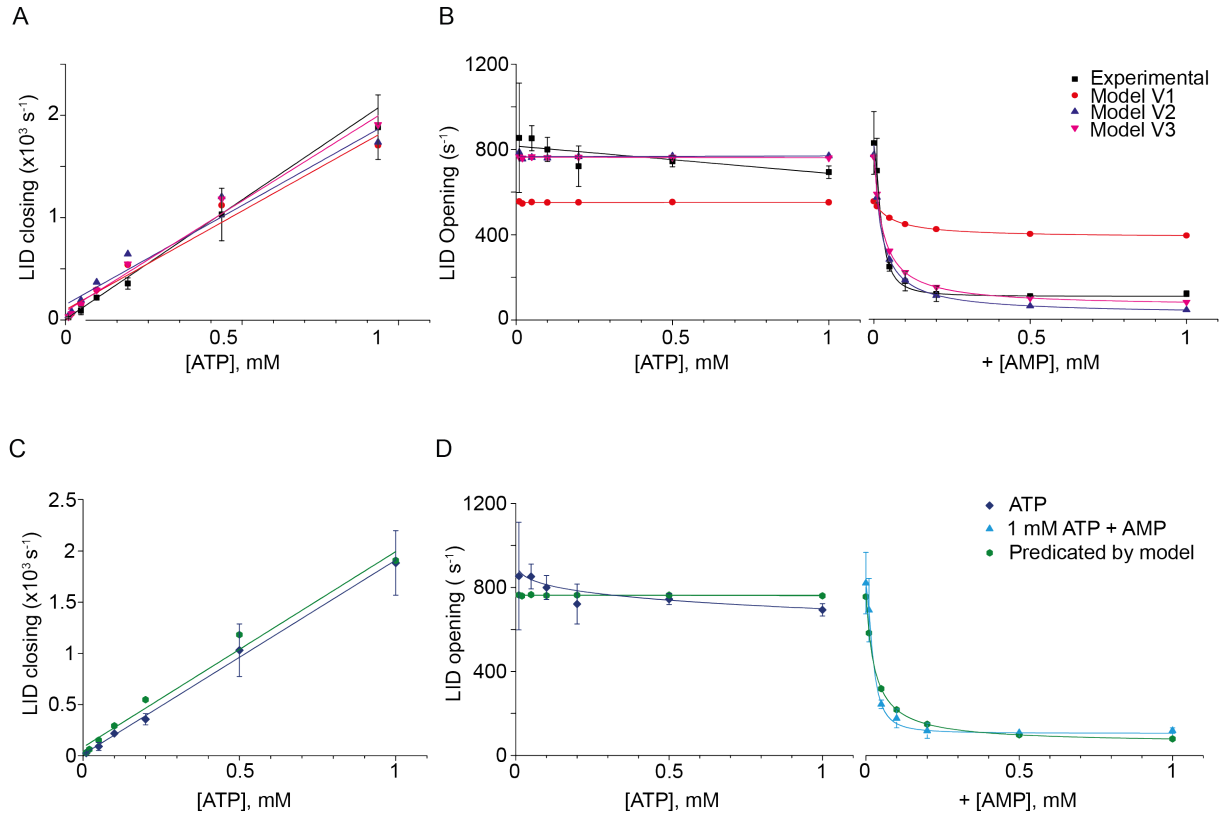

Supplementary Figure 18 – Experimental and predicted kinetic values for binding of ATP and ATP + AMP to AK_2+. (A) Frequency of LID closing - measured as the inverse of dwell times – at increasing ATP concentrations for different models and the experimental values. The lines indicate a linear fit. **(B)** LID opening frequency at increasing ATP concentrations for the different models tested and the experimental values. The lines are fitting the data to a Hill equation. **(C)** Frequency of LID closing - measured as the inverse of dwell times – at increasing ATP concentrations for model V3 and the experimental values. The lines indicate a linear fit. **(D)** LID opening frequency at increasing ATP concentrations for model V3 and the experimental values. The lines are fitting the data to a Hill equation.The measurements were performed in 400 mM KCl, 15 mM Tris, 2 mM $MgCl_2$, pH 7.5 at room temperature (22°C) applying -90 mV (*trans*) and sampling at 50 kHz with a 10 kHz Bessel-low filter, additionally digitally filtered with a 2 kHz Gaussian low-pass filter. Ligands were added to the *trans* chamber and the enzyme to the *cis* chamber. Error bars in all graphs represents the standard deviation of the mean between independent experiments (N = 3).

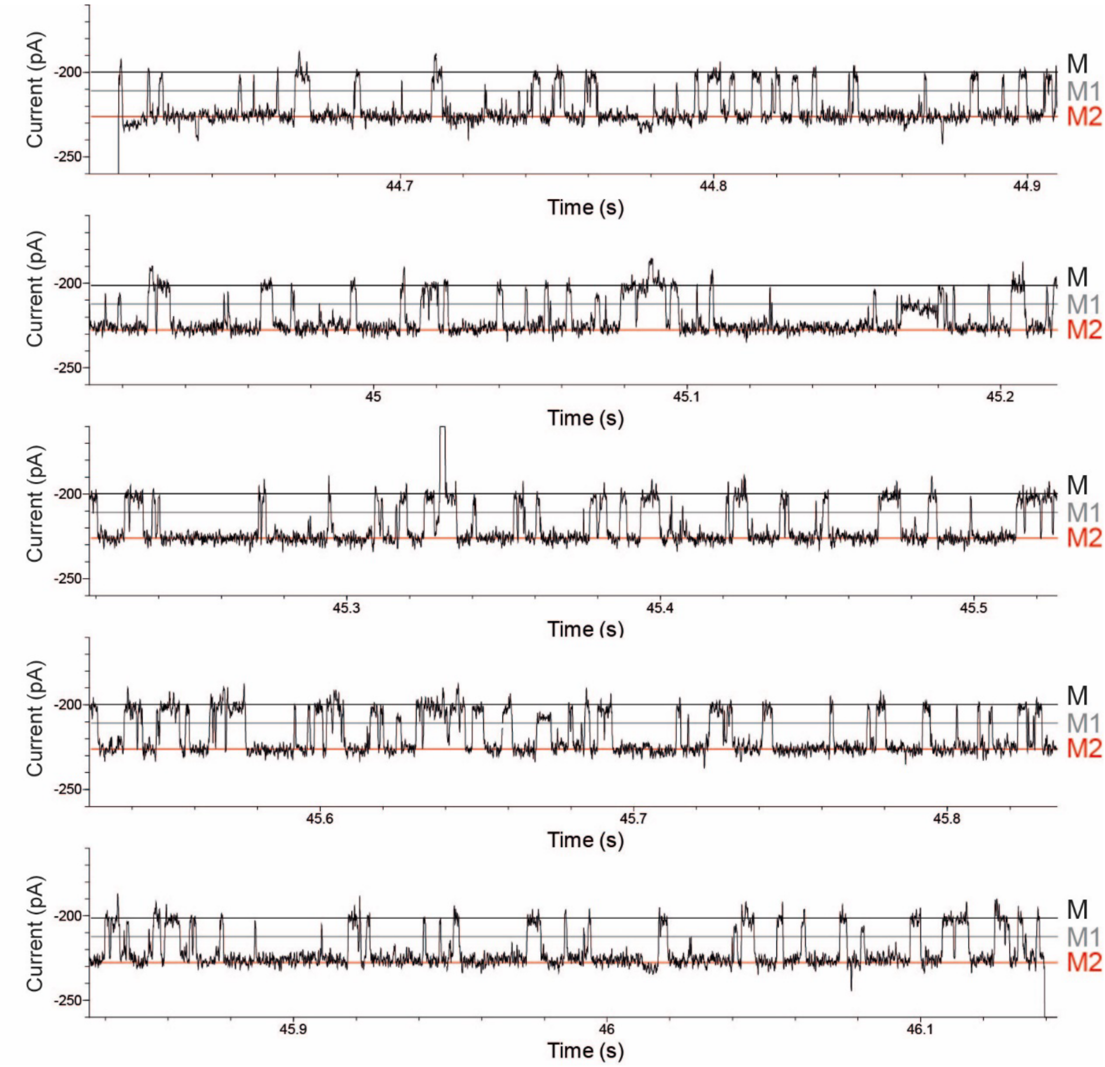

Supplementary Figure 19. Continuous recording of AK_2+ conformational changes during the binding of ADP type I. ADP (100 µM) was added to the *trans* chamber. M and M1 represent the open conformation (black and grey line) and M2 the protein with the LID domain closed (red line). The measurements were performed in 400 mM KCl, 15 mM Tris, 2 mM $MgCl_2$, pH 7.5 at room temperature (22°C) applying -90 mV (*trans*) and sampling at 50 kHz with a 10 kHz Bessel-low filter, additionally digitally filtered with a 2 kHz Gaussian low-pass filter.

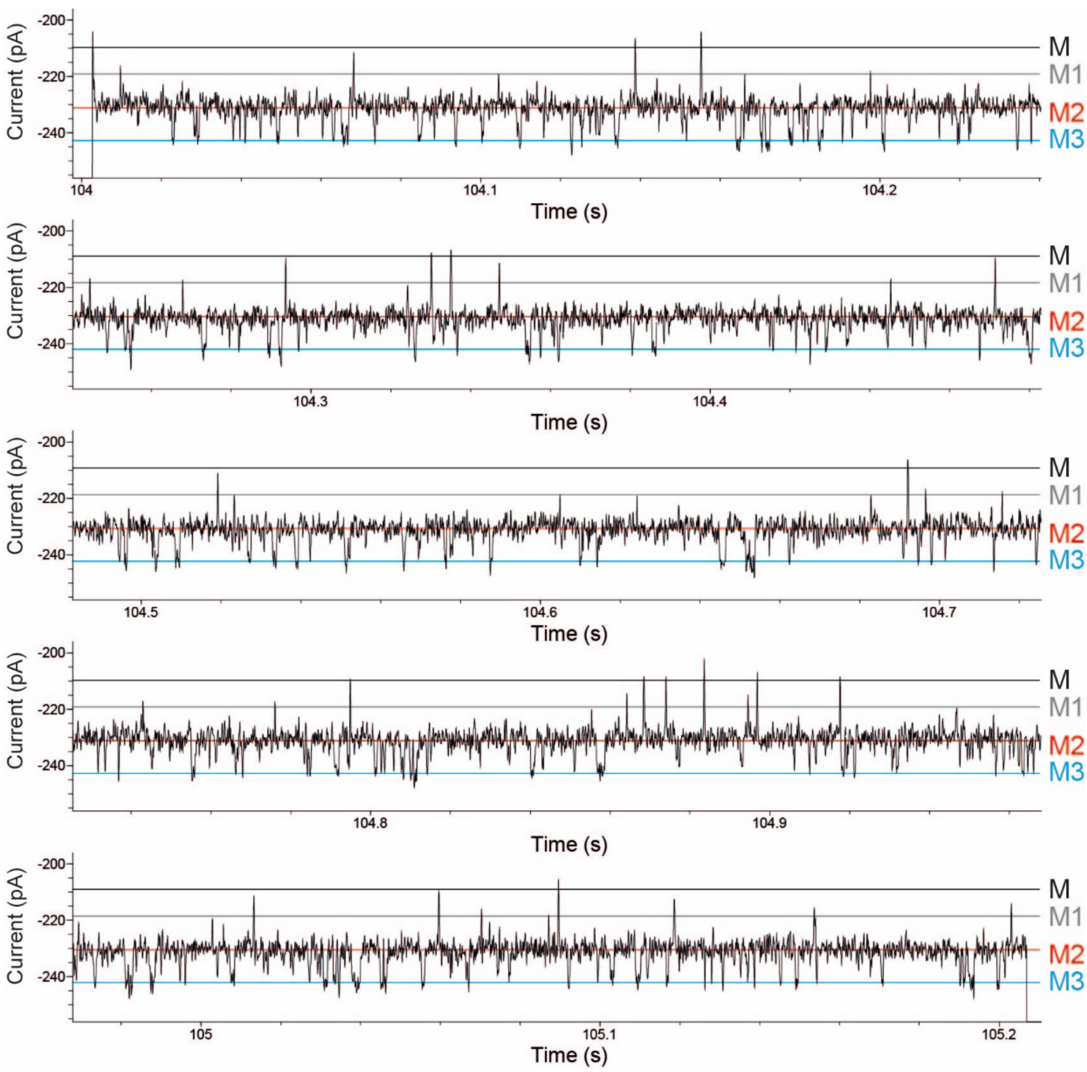

Supplementary Figure 20. Continuous recording of AK_2+ conformational changes during the binding of ADP type I. ADP (1 mM) was added to the *trans* chamber. M and M1 represent the open conformation (black and grey line), M2 the protein with the LID domain closed (red line) and M3 with both the LID and NMP domain closed (blue line). The measurements were performed in 400 mM KCl, 15 mM Tris, 2 mM $MgCl_2$, pH 7.5 at room temperature (22°C) applying -90 mV (*trans*) and sampling at 50 kHz with a 10 kHz Bessel-low filter, additionally digitally filtered with a 2 kHz Gaussian low-pass filter.

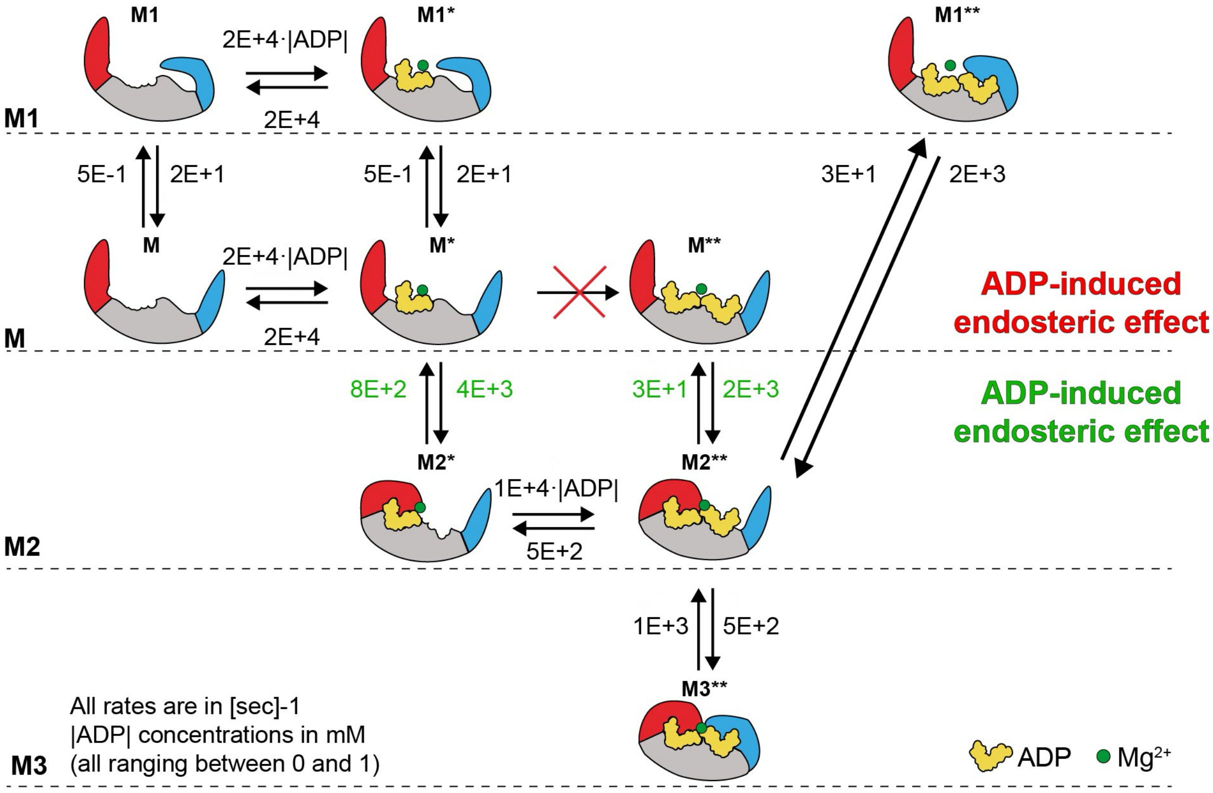

Supplementary Figure 21. Kinetic model for the endosteric closing of the LID and NMP domain. The model includes the four observed states for the LID and NMP domain, and the hidden ligand-bound states. The ADP-induced endosteric effect was implemented by removing the M* → M** transition. The ADP-induced endosteric effect was implemented by allowing the M* ↔ M2* and M** ↔ M2** states to have different rates. The rates were retrieved by fitting the kinetic data to a Hidden Markov Model (HMM) based on the four observable states and using different ligand concentration. Confidence intervals for the estimated rates are given in Supplementary Table 11.

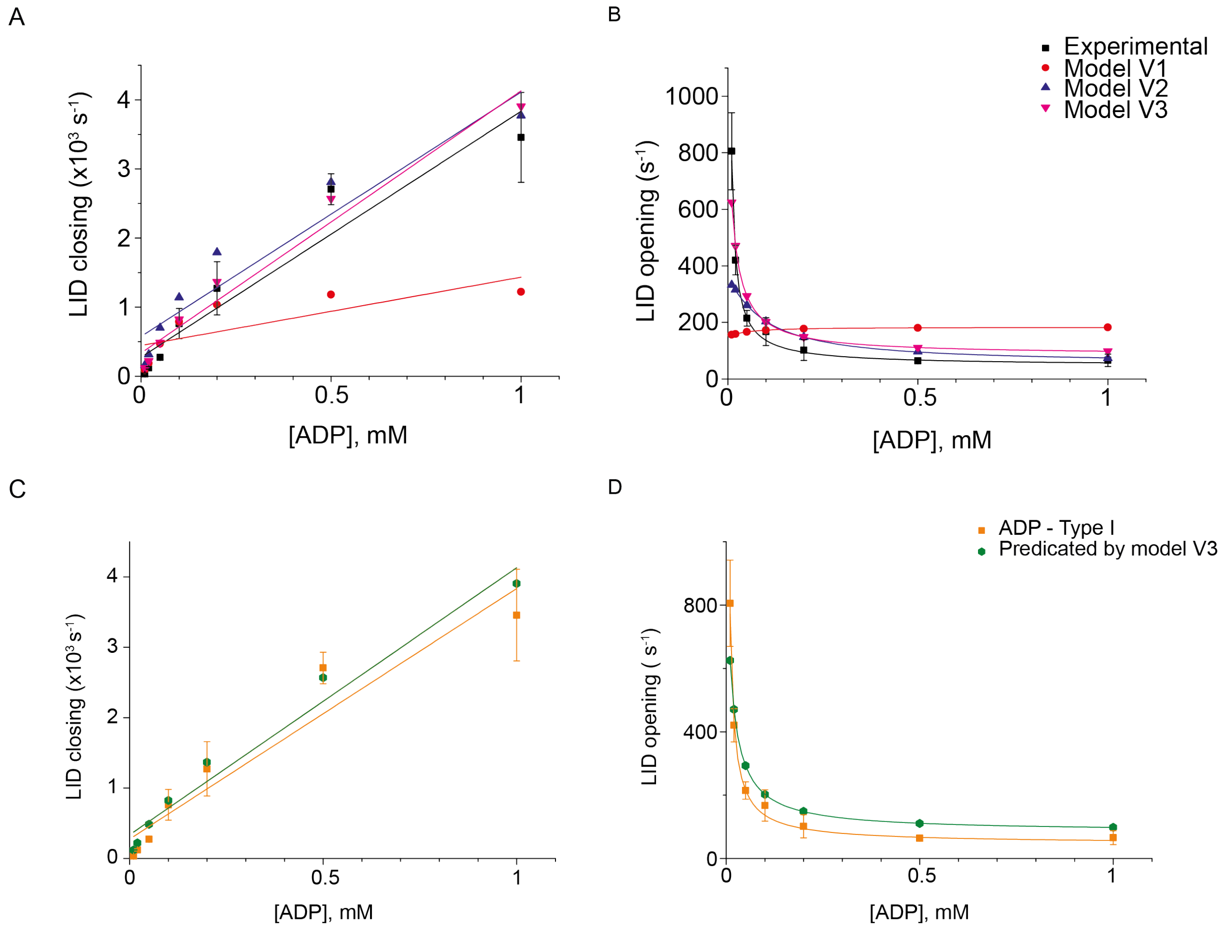

Supplementary Figure 22 – Experimental and predicted kinetic values for binding of ADP type I to AK_2+. (A) Frequency of LID closing - measured as the inverse of dwell times – at increasing ADP concentrations for the different models. The lines indicate a linear fit. **(B)** LID opening frequency at increasing ADP concentrations for the different models. The lines are fitting the data to a Hill equation. **(C)** Frequency of LID closing - measured as the inverse of dwell times – at increasing ADP concentrations for the model V3 and the experimental values. The lines indicate a linear fit. **(D)** LID opening frequency at increasing ADP concentrations for model V3 and the experimental values. The lines are fitting the data to a Hill equation. The measurements were performed in 400 mM KCl, 15 mM Tris, 2 mM $MgCl_2$, pH 7.5 at room temperature (22°C) applying -90 mV (*trans*) and sampling at 50 kHz with a 10 kHz Bessel-low filter, additionally digitally filtered with a 2 kHz Gaussian low-pass filter. Ligands were added to the *trans* chamber and the enzyme to the *cis* chamber. Error bars in all graphs represents the standard deviation of the mean between independent experiments (N = 3).

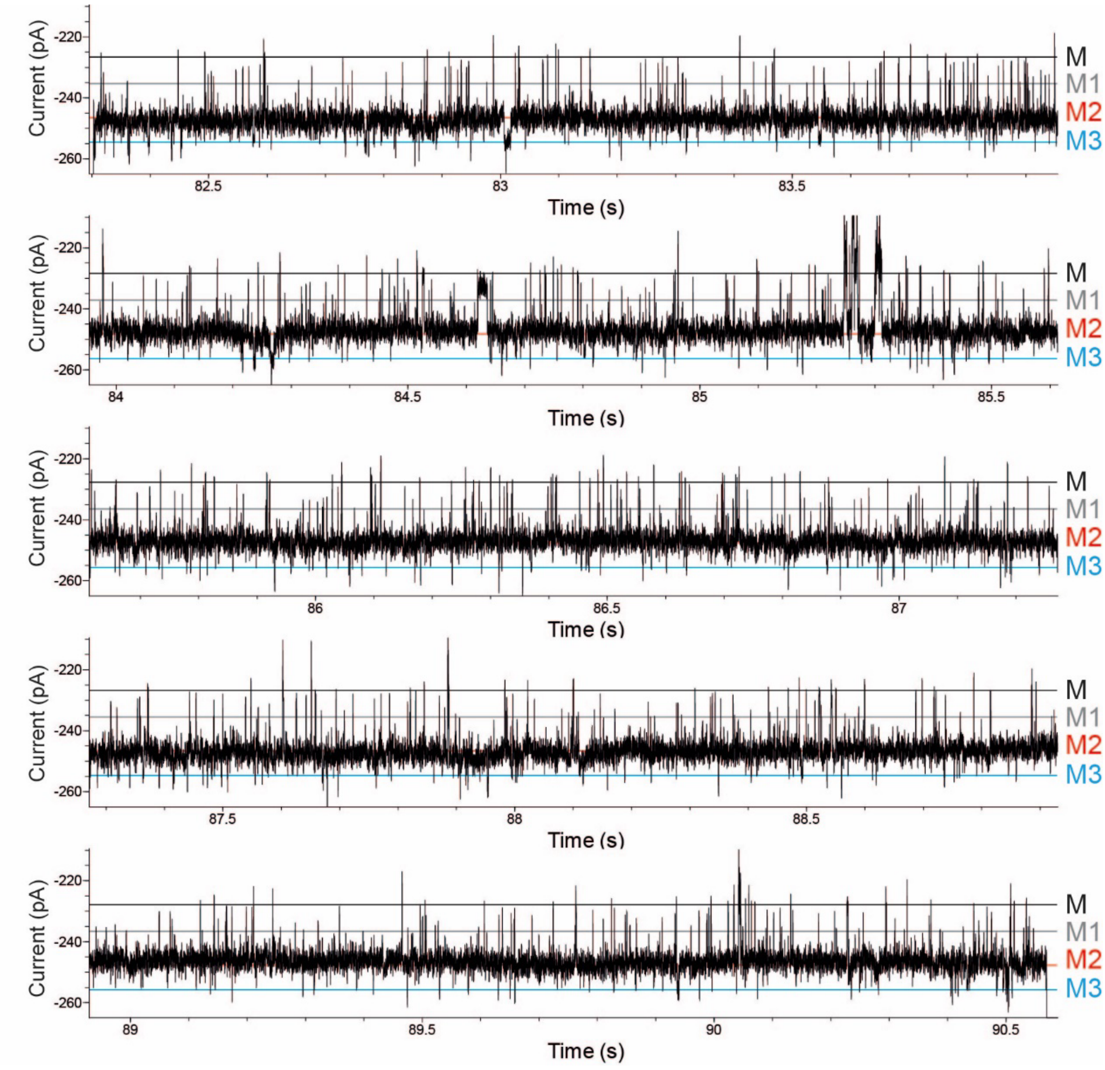

Supplementary Figure 23. Continuous recording of AK_2+ conformational changes during the binding of ADP type I without Mg^{2+}. ADP (1 mM) was added to the *trans* chamber. M and M1 represent the open conformation (black and grey line), M2 the protein with the LID domain closed (red line) and M3 with both the LID and NMP domain closed (blue line). The measurements were performed in 400 mM KCl, 15 mM Tris, 100 µM EDTA, pH 7.5 at room temperature (22°C) applying -90 mV (*trans*) and sampling at 50 kHz with a 10 kHz Bessel-low filter, additionally digitally filtered with a 2 kHz Gaussian low-pass filter.

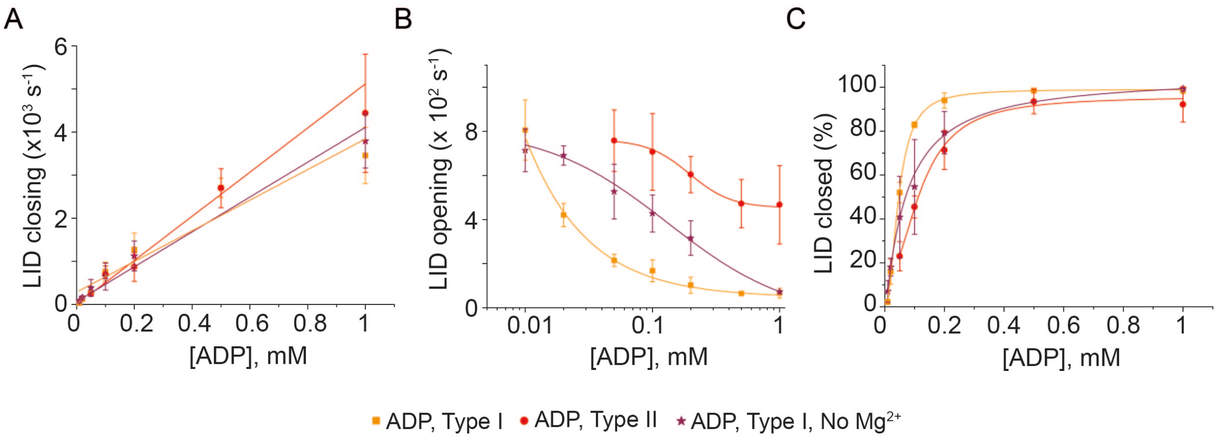

Supplementary Figure 24. AK_2+ kinetics in the ClyA-AS nanopore upon binding of ADP without magnesium. (a) LID closing frequency - measured as the inverse of dwell times – at increasing ATP concentrations. The lines indicate a linear fit. **(b)** LID opening frequency at increasing ADP concentrations. The lines indicate a Hill equation fit. **(C)** Percentage of the closed LID at increasing ligand concentrations. The lines indicate a Hill equation fit. The measurements were performed in 400 mM KCl, 15 mM Tris, pH 7.5 at room temperature (22°C) applying -90 mV (*trans*) and sampling at 50 kHz with a 10 kHz Bessel-low filter, additionally digitally filtered with a 2 kHz Gaussian low-pass filter. Ligands were added to the *trans* chamber and the enzyme to the *cis* chamber. Error bars in all graphs represents the standard deviation of the mean between independent experiments (N = 3).

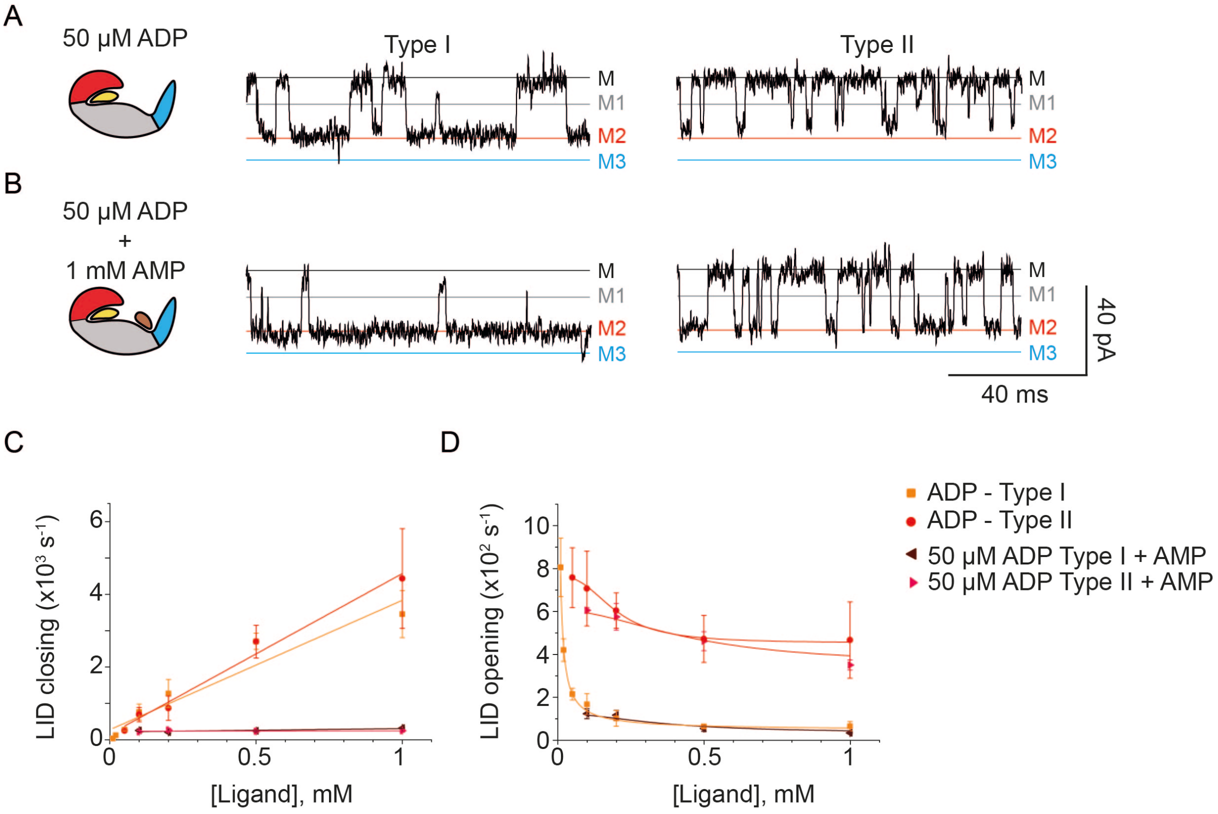

Supplementary Figure 25: Ligand binding behavior of AK_2+ with ADP and AMP. (A) Expansions of typical ionic current blockades provoked by the capture of a single AK_2+ molecule in the presence of 50 μM ADP and **(B)** in the presence of 50 μM ADP and 1 mM AMP (both *trans*). **(C)** LID closing frequency - measured as the inverse of dwell times – at increasing ATP concentrations. The lines indicate a linear fit. **(D)** LID opening frequency at increasing ADP concentrations. The lines indicate a Hill equation fit. The measurements were performed in 400 mM KCl, 15 mM Tris, 2 mM $MgCl_2$, pH 7.5 at room temperature (22°C) applying -90 mV (*trans*) and sampling at 50 kHz with a 10 kHz Bessel-low filter, additionally digitally filtered with a 2 kHz Gaussian low-pass filter. Error bars for all graphs represents the standard deviation of the mean between independent experiments (N = 3).

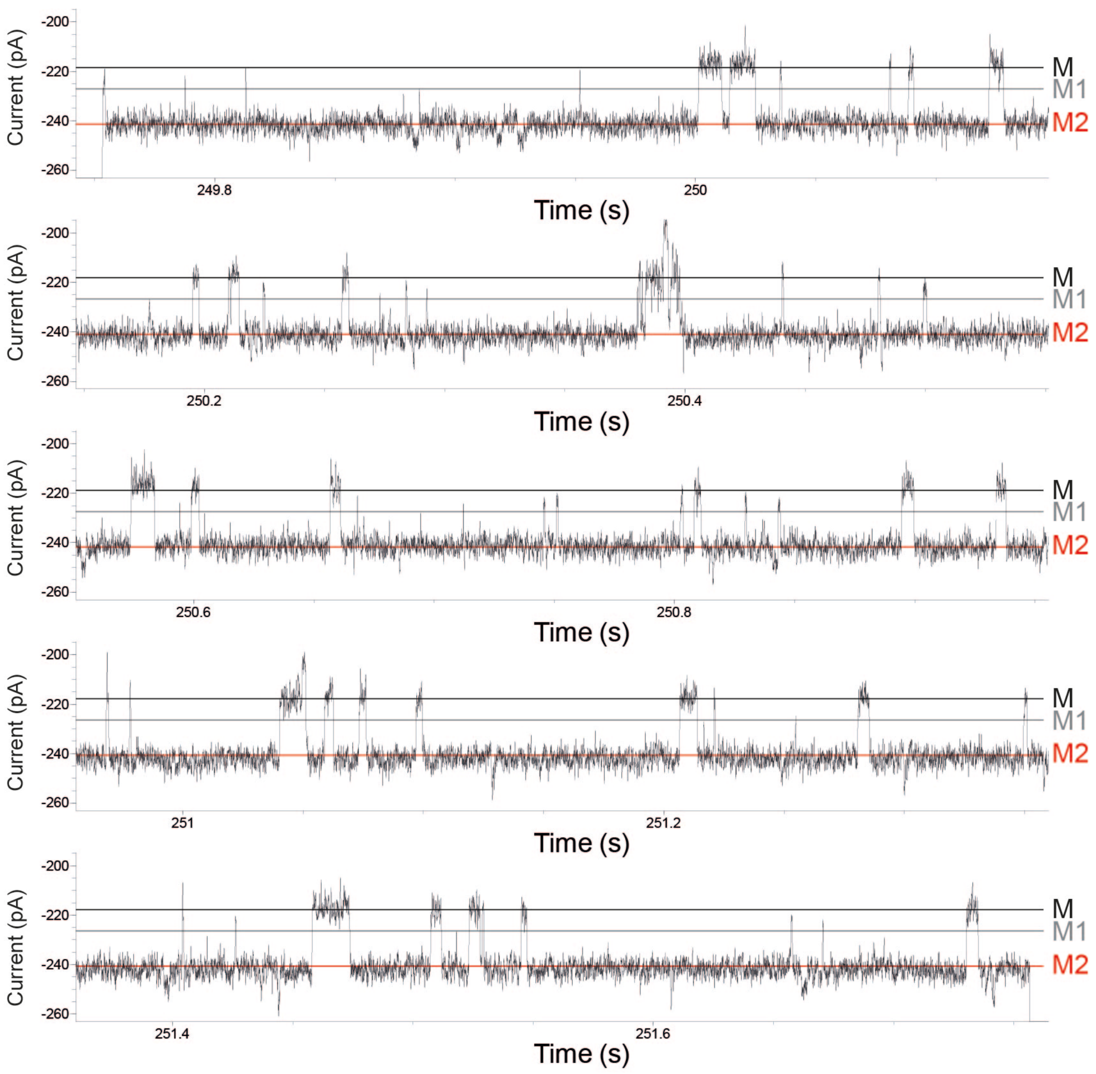

Supplementary Figure 26: Continuous recording of AK_2+ conformational changes during the binding of ADP type I and AMP. ADP (50 µM) and AMP (1 mM) were added to the *trans* chamber. M and M1 represent the open conformation (black and grey line) and M2 the protein with the LID domain closed (red line). The measurements were performed in 400 mM KCl, 15 mM Tris, 2 mM $MgCl_2$, pH 7.5 at room temperature (22°C) applying -90 mV (*trans*) and sampling at 50 kHz with a 10 kHz Bessel-low filter, additionally digitally filtered with a 2 kHz Gaussian low-pass filter.

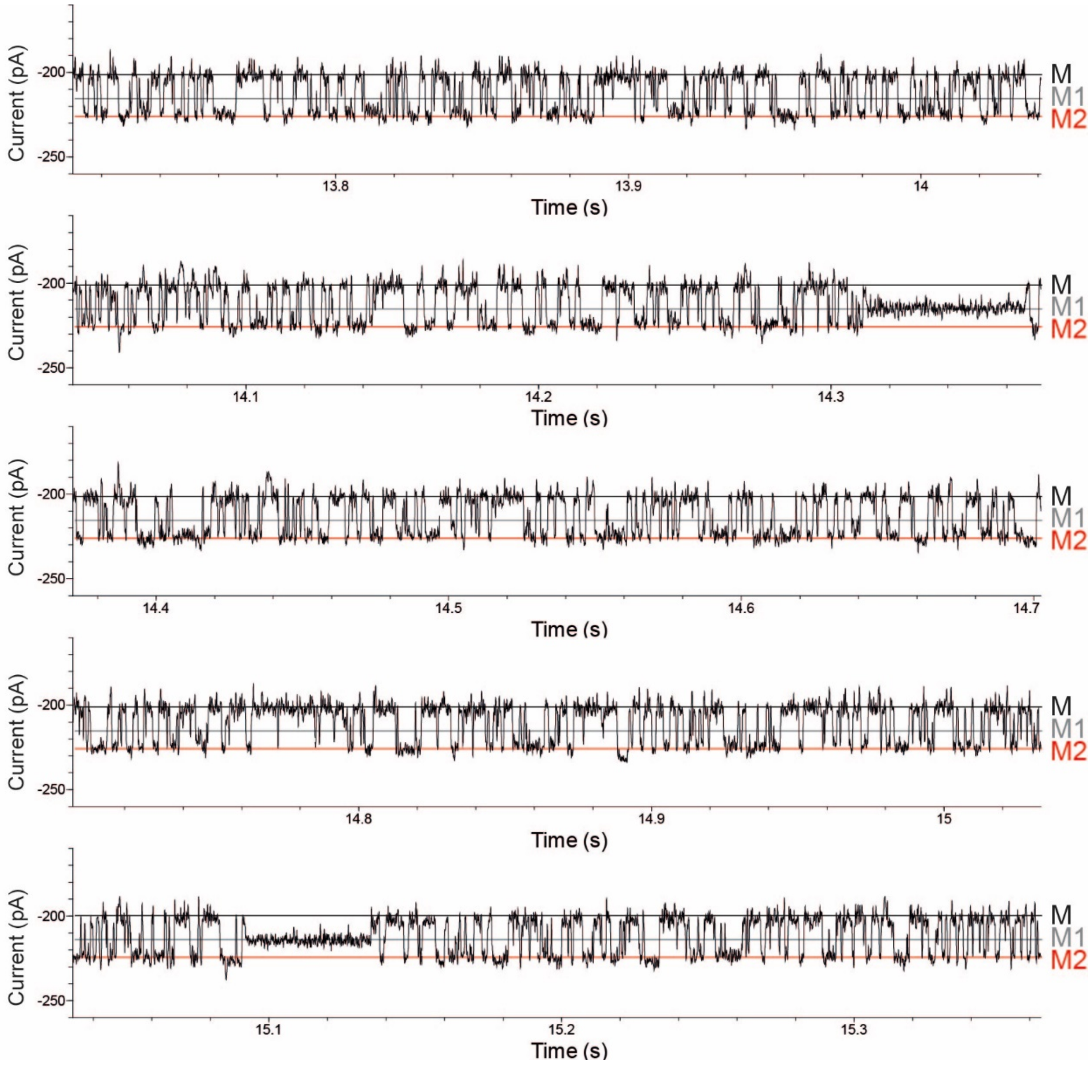

Supplementary Figure 27. Continuous recording of AK_2+ conformational changes during the binding of ADP type II. ADP (100 µM) was added to the *trans* chamber. M and M1 represent the open conformation (black and grey line) and M2 the protein with the LID domain closed (red line). The measurements were performed in 400 mM KCl, 15 mM Tris, 2 mM $MgCl_2$, pH 7.5 at room temperature (22°C) applying -90 mV (*trans*) and sampling at 50 kHz with a 10 kHz Bessel-low filter, additionally digitally filtered with a 2 kHz Gaussian low-pass filter.

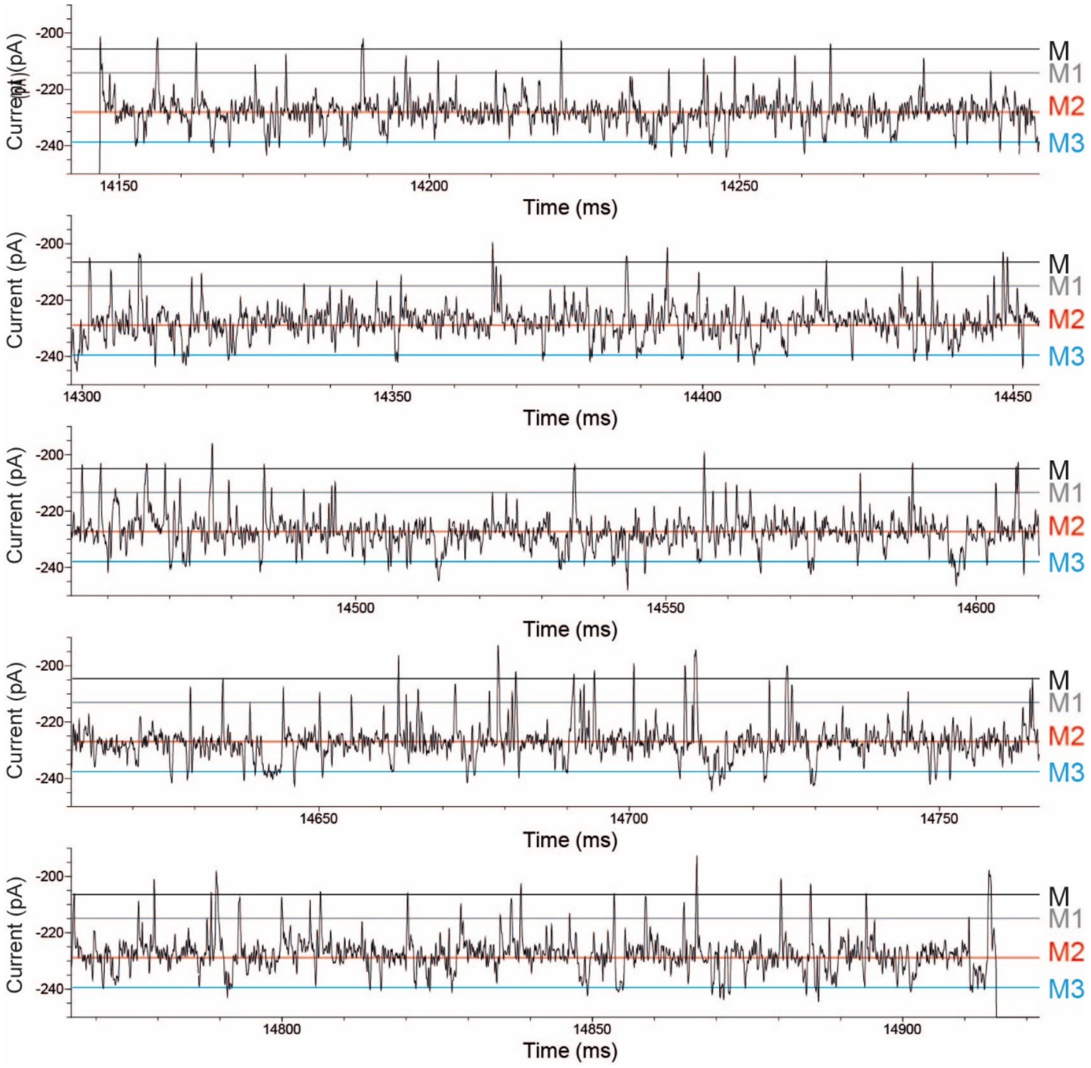

Supplementary Figure 28. Continuous recording of AK_2+ conformational changes during the binding of ADP type II. ADP (1 mM) was added to the *trans* chamber. M and M1 represent the open conformation (black and grey line), M2 the protein with the LID domain closed (red line) and M3 with both the LID and NMP domain closed (blue line). The measurements were performed in 400 mM KCl, 15 mM Tris, 2 mM $MgCl_2$, pH 7.5 at room temperature (22°C) applying -90 mV (*trans*) and sampling at 50 kHz with a 10 kHz Bessel-low filter, additionally digitally filtered with a 2 kHz Gaussian low-pass filter.

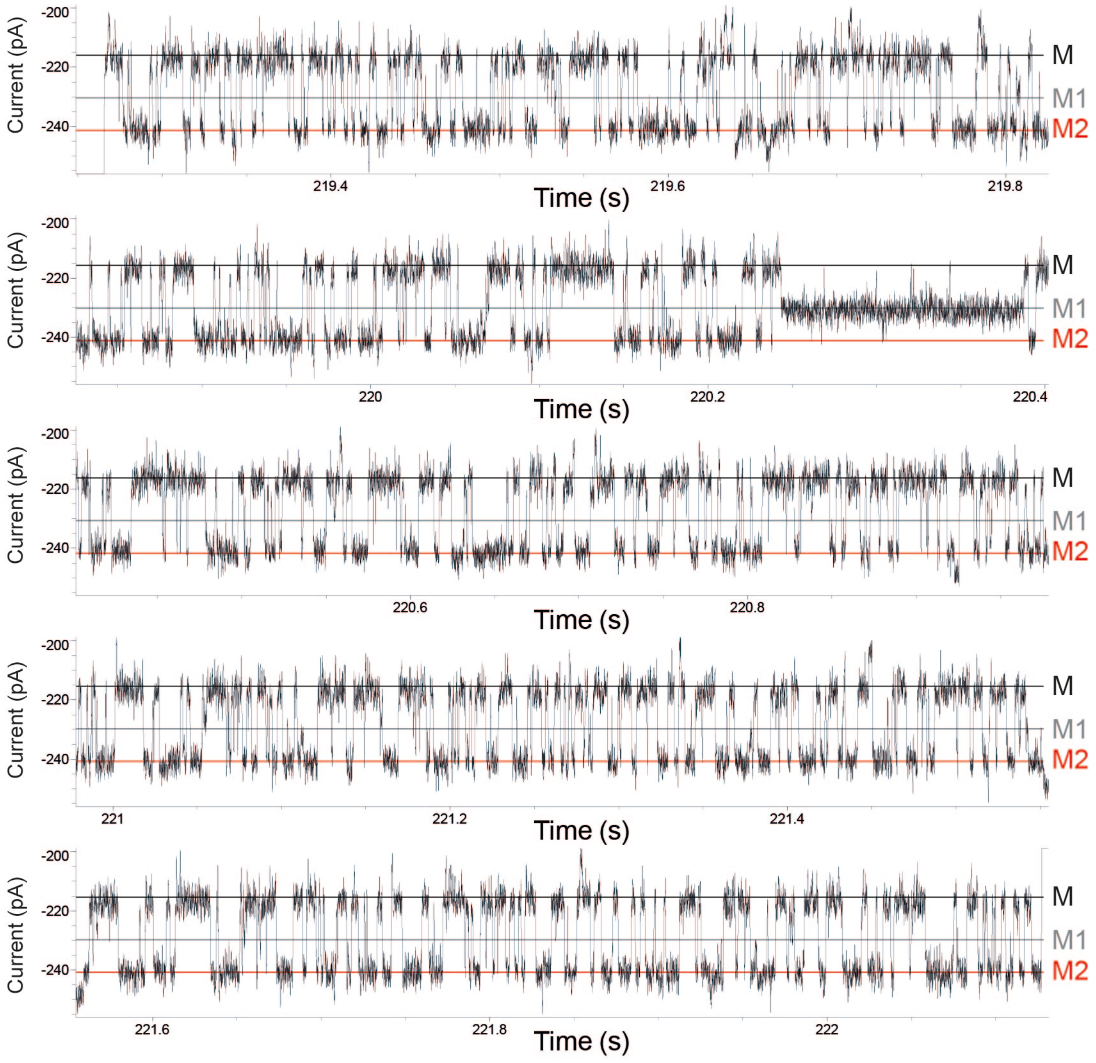

Supplementary Figure 29: Continuous recording of AK_2+ conformational changes during the binding of ADP type II and AMP. ADP (50 µM) and AMP (1 mM) were added to the *trans* chamber. M and M1 represent the open conformation (black and grey line) and M2 the protein with the LID domain closed (red line). The measurements were performed in 400 mM KCl, 15 mM Tris, 2 mM $MgCl_2$, pH 7.5 at room temperature (22°C) applying -90 mV (*trans*) and sampling at 50 kHz with a 10 kHz Bessel-low filter, additionally digitally filtered with a 2 kHz Gaussian low-pass filter.

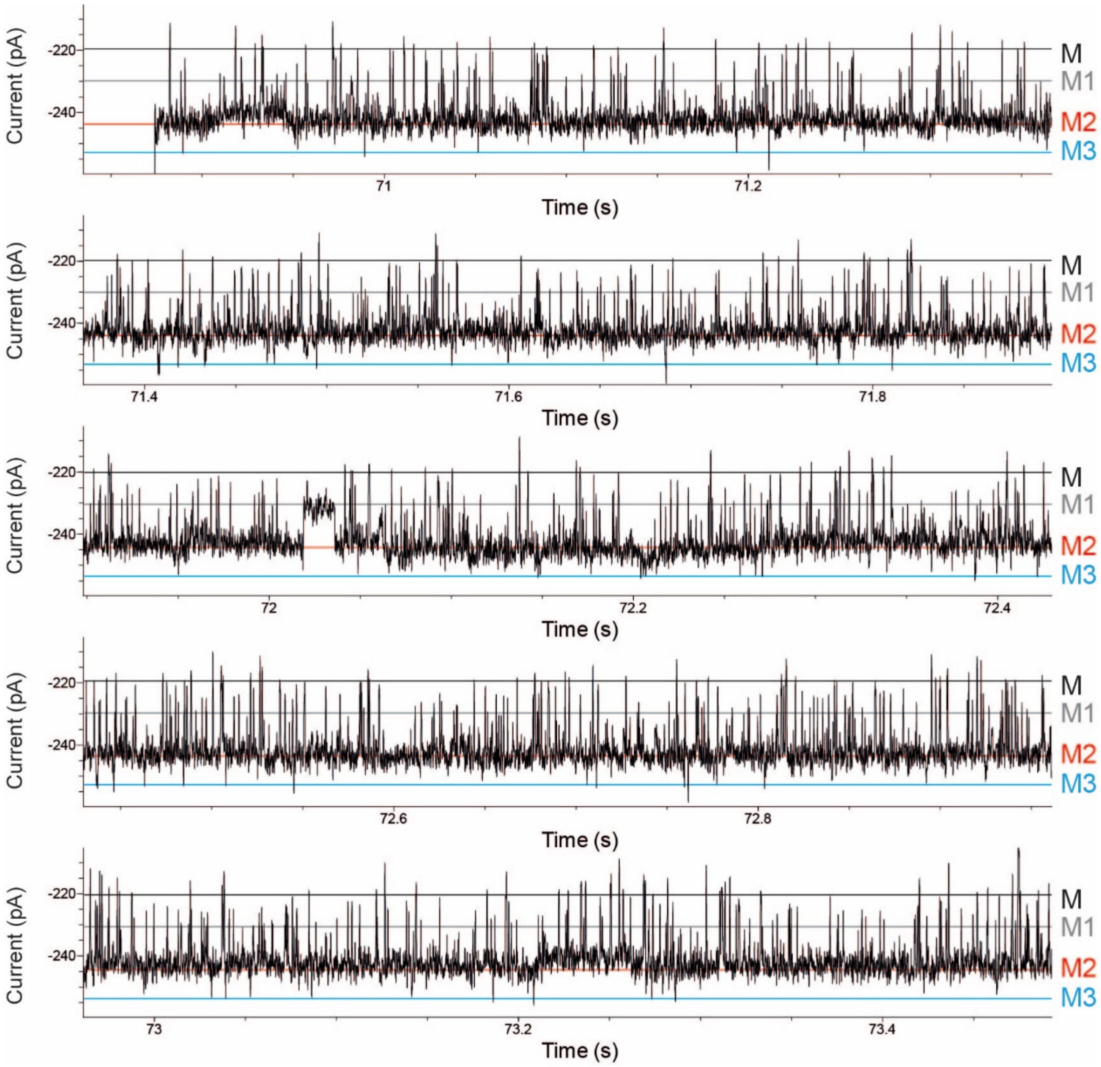

Supplementary Figure 30. Continuous recording of AK_2+ conformational changes during the binding of ADP type II without Mg^{2+}. ADP (1 mM) was added to the *trans* chamber. M and M1 represent the open conformation (black and grey line), M2 the protein with the LID domain closed (red line) and M3 with both the LID and NMP domain closed (blue line). The measurements were performed in 400 mM KCl, 15 mM Tris, 100 µM EDTA, pH 7.5 at room temperature (22°C) applying -90 mV (*trans*) and sampling at 50 kHz with a 10 kHz Bessel-low filter, additionally digitally filtered with a 2 kHz Gaussian low-pass filter.

Supplementary Table 1. Results of AK bulk activity assay.

	K_M (μM)	V_{max} (μM/min)	Hill coefficient	K_{cat} (s^{-1})
AK-WT	261±106	54±14	1.6±0.6	30±8
AK_2+	195±8	34±1	2.1±0.1	18.9±0.6

Supplementary Table 2: Table with current blockades of AK_2+ upon addition of ligand. Errors are given as standard deviation of the mean between independent pores (*N* = 3) All current levels were collected in 400 mM KCl, 15 mM Tris, 2 mM $MgCl_2$, pH 7.5 at room temperature (22°C) applying -90 mV (*trans*).

Level	Ligand	I_{res} (%)	ΔI_{res} (%)	ΔI_{res} (pA)
M1		49.2 ± 0.1	3.2 ± 0.3	13.6 ± 0.7
M2	**AMP**	51.8 ± 0.1	5.7 ± 0.2	24.3 ± 0.9
M2	**ATP**	52.0 ± 0.1	5.7 ± 0.4	25.4 ± 1.9
M3	**ATP + AMP**	54.5 ± 0.2	7.3 ± 0.1	32.1 ± 1.4
M2	**ADP**	51.8 ± 0.2	5.4 ± 0.2	23.8 ± 0.6
M3	**ADP**	54.3 ± 0.2	7.5 ± 0.6	33.2 ± 0.3
M2 Type I	**ADP**	51.5 ± 0.4	5.1 ± 0.2	22.3 ± 0.9
M3 Type II	**ADP**	53.5 ± 0.8	7.8 ± 1.8	31.4 ± 3.8
M<br>M1<br>M2<br>M3<br>M4	**Ap5A**	46.4 ± 0.2<br>48.7 ± 0.2<br>51.0 ± 0.1<br>53.8 ± 0.1<br>56.6 ± 0.2	/<br>2.3 ± 0.2<br>4.6 ± 0.1<br>7.4 ± 0.1<br>10.2 ± 0.1	/<br>10.4 ± 0.4<br>20.6 ± 0.8<br>32.5 ± 1.0<br>45.1 ± 0.6

Supplementary Table 3. AK_2+ binding behavior in the ClyA-AS nanopore with ATP with their individual values obtained in this study. Errors are given as standard deviation of the mean between independent pores (*N* ≥ 3). All current levels were collected in 400 mM KCl, 15 mM Tris, 2 mM $MgCl_2$ (unless omitted), pH 7.5 at room temperature (22°C) applying -90 mV (*trans*).

**Concentration ligand (µM) ATP**	**On rate (s^{-1})**	**Off rate (s^{-1})**	**Lid closed (%)**
10	25.6 ± 7.2	854.3 ± 256.5	1.0 ± 0.6
50	90.8 ± 38.9	852.5 ± 59.1	6.4 ± 2.4
100	218.0 ± 7.9	799.8 ± 57.0	18.3 ± 4.2
200	356.2 ± 54.8	721.4 ± 94.9	29.2 ± 6.4
500	1031.0 ± 257.0	744.5 ± 25.8	65.0 ± 3.6
1000	1883.9 ± 315.2	693.4 ± 29.6	75.9 ± 7.3
**Concentration ligand (µM) ATP**	**On rate (s^{-1}) No Mg^{2+}**	**Off rate (s^{-1}) No Mg^{2+}**	**Lid closed (%) No Mg^{2+}**
10	-	-	-
50	77.6 ± 42.2	4165.0 ± 439.1	0.6 ± 0.1
100	221.8 ± 38.1	4797.4 ± 198.4	1.6 ± 0.1
200	339.9 ± 88.1	4312.5 ± 156.6	2.8 ± 0.7
500	782.3 ± 195.5	3929.0 ± 80.6	8.8 ± 4.5
1000	1461.5 ± 99.1	3537.6 ± 96.9	24.4 ± 7.4
**Concentration ligand (µM) ATP**	**On rate (s^{-1}) R156A**	**Off rate (s^{-1}) R156A**	**Lid closed (%) R156A**
10	20.3 ± 4.4	874.9 ± 74.9	1.2 ± 0.5
50	-	-	-
100	158.8 ± 32.0	764.6 ± 36.5	14.4 ± 3.0
200	-	-	-
500	-	-	-
1000	1290.7 ± 203.8	800.1 ± 27.4	62.3 ± 3.2

Supplementary Table 4. AK_2+ binding behavior in the ClyA-AS nanopore with ATP with their individual values obtained in this study. Errors are given as standard deviation of the mean between independent pores (*N* ≥ 3). All current levels were collected in 250 mM KCl, 15 mM Tris, 2 mM $MgCl_2$, pH 7.5 at room temperature (22°C) applying -90 mV (*trans*).

**Concentration ligand (µM) ATP**	**On rate (s^{-1})**	**Off rate (s^{-1})**	**Lid closed (%)**
10	53.8 ± 11.9	592.2 ± 26.6	9.3 ± 1.5
50	237.7 ± 17.5	569.9 ± 17.5	32.7 ± 1.7
100	426.3 ± 76.8	573.1 ± 48.0	48.5 ± 5.5
200	734.8 ± 17.8	554.6 ± 16.3	62.6 ± 0.5
500	1658.6 ± 105.4	448.1 ± 21.3	83.1 ± 0.4

1000	3054.1 ± 110.3	393.1 ± 5.3	91.5 ± 0.6

Supplementary Table 5. AK_2+ binding behavior in the ClyA-AS nanopore with 1 mM ATP and AMP with their individual values obtained in this study. Errors are given as standard deviation of the mean between independent pores (*N* ≥ 3). All current levels were collected in 400 mM KCl, 15 mM Tris, 2 mM $MgCl_2$ (unless omitted), pH 7.5 at room temperature (22°C) applying -90 mV (*trans*).

**Concentration ligand (µM) 1 mM ATP + [AMP]**	**On rate LID (s^{-1})**	**Off rate LID (s^{-1})**	**Off rate NMP domain (s^{-1})**	**Lid closed (%)**
0	1380.7 ± 569.2	825.2 ± 146.9	-	75.4 ± 7.6
10	1816.8 ± 227.0	695.8 ± 151.5	-	78.0 ± 5.6
20	-	-	-	-
50	2066.0 ± 310.12	245.7 ± 20.6	4436.9 ± 5634	92.9 ± 2.3
100	2082.2 ± 204.1	177.9 ± 45.1	4123.3 ± 625.9	96.7 ± 1.0
200	2196.7 ± 131.8	118.9 ± 36.3	3265.4 ± 723.8	98.6 ± 0.7
500	2523.8 ± 241.9	109.0 ± 1.8	3661.1 ± 220.7	99.4 ± 0.1
1000	2423.3 ± 649.9	119.6 ± 13.0	3172.0 ± 257.7	99.5 ± 0.1
**Concentration ligand (µM) 1 mM ATP + [AMP]**	**On rate (s^{-1}) No Mg^{2+}**	**Off rate (s^{-1}) No Mg^{2+}**	**Off rate (s^{-1}) NMP domain No Mg^{2+}**	**Lid closed (%) No Mg^{2+}**
0	1461.5 ± 99.1	3537.6 ± 96.9	-	24.2 ± 7.4
10	-	-	-	-
20	1714.3 ± 100.7	418.8 ± 244.6<br>2957.7 ± 656.4	-	36.7 ± 10.9
50	1819.1 ± 146.9	287.0 ± 52.6<br>2918.2 ± 415.4	-	45.6 ± 6.1
100	1784.2 ± 100.2	131.6 ± 46.2<br>2854.6 ± 422.7	-	52.5 ± 10.3
200	1817.1 ± 25.2	107.2 ± 15.5<br>2785.9 ± 231.1	-	52.0 ± 3.6
500	1696.9 ± 47.4	61.5 ± 16.4<br>2558.9 ± 717.9	-	66.9 ± 13.7
1000	1768.3 ± 185.4	40.3 ± 18.0<br>2000.6 ± 546.8	-	79.5 ± 10.0
**Concentration ligand (µM) 1 mM ATP + [AMP]**	**On rate (s^{-1}) R156A**	**Off rate (s^{-1}) R156A**	**Off rate (s^{-1}) NMP domain R156A**	**Lid closed (%) R156A**
0	1290.7 ± 203.8	800.1 ± 27.4	-	62.3 ± 3.2
10	1276.0± 141.6	681.0 ± 37.6	-	66.7 ± 1.3
20	-	-	-	-
50	-	-	-	-
100	1452.6 ± 95.6	626.8 ± 95.4	-	70.9 ± 2.5
200	-	-	-	-
500	-	-	-	-

1000	1513.4 ± 178.1	418.1 ± 11.2	-	80.4 ± 1.4

Supplementary Table 6. AK_2+ binding behavior in the ClyA-AS nanopore with ADP with their individual values obtained in this study. Errors are given as standard deviation of the mean between independent pores (*N* ≥ 3). All current levels were collected in 400 mM KCl, 15 mM Tris, 2 mM $MgCl_2$ (unless omitted), pH 7.5 at room temperature (22°C) applying -90 mV (*trans*).

**Concentration ligand (µM) ADP**	**On rate Type I (s^{-1})**	**Off rate Type I (s^{-1})**	**Lid closed Type I (%)**
10	32.6 ± 13.3	805.8 ± 136.3	2.1 ± 1.3
20	121.2 ± 18.4	420.5 ± 52.5	15.9 ± 5.7
50	272.2 ± 30.1	214.8 ± 27.6	52.0 ± 4.7
100	762.2 ± 219.2	167.5 ± 49.4	82.9 ± 1.3
200	1272.0 ± 385.7	101.9 ± 36.5	93.9 ± 3.4
500	2706.7 ± 223.4	64.3 ± 9.2	98.5 ± 0.7
1000	3456.9 ± 650.1	65.9 ± 22.0	98.8 ± 1.6
**Concentration ligand (µM) ADP**	**On rate Type I (s^{-1}) No Mg^{2+}**	**Off rate Type I (s^{-1}) No Mg^{2+}**	**Lid closed Type I (%) No Mg^{2+}**
10	78.7 ± 38.0	713.6 ± 96.7	7.0 ± 0.5
20	162.2 ± 36.8	690.6 ± 43.8	18.0 ± 4.1
50	385.6 ± 194.0	526.6 ± 123.8	40.8 ± 18.6
100	648.6 ± 313.3	427.5 ± 84.0	54.6 ± 21.6
200	1125.0 ± 347.2	315.8 ± 78.3	79.3 ± 9.6
500	-	-	-
1000	3791.5 ± 624.3	73.0 ± 1.5	99.3 ± 0.2
**Concentration ligand (µM) ADP**	**On rate Type II (s^{-1})**	**Off rate Type II (s^{-1})**	**Lid closed Type II (%)**
10	-	-	-
20	-	-	-
50	246.8 ± 35.4	757.8 ± 139.5	23.0 ± 6.7
100	693.9 ± 198.4	706.7 ± 174.1	45.5 ± 5.0
200	868.7 ± 334.9	604.2 ± 82.5	71.4 ± 8.8
500	2697.1 ± 450.4	471.9 ± 109.6	93.5 ± 5.6
1000	4434.7 ± 1372.5	466.9 ± 177.6	92.1 ± 8.0

Supplementary Table 7. Comparison of dissociation constants, and the opening and closing rates for AK from other studies.

Ligand	K_m (μM)	V_{max} (units/mg)	k_{cat} (s^{-1})	k_{cat} / K_m (s^{-1} μM^{-1})	K_D (μM)	$k_{opening}$ (LID) (s^{-1})	$k_{closing}$ (LID) (s^{-1})
ATP (+mg2+)	71[7]<br>42[8]<br>144[9]<br>60[3]	780[7]<br>1050[9]	305[7]	4.3[7]<br>0.16[8]	85[7]<br>170[8]		
ATP					35[7]<br>44[8]<br>50[10]		
AMP (+mg2+)	33[9]	1038[9]					
AMP	26[7]<br>98[8]<br>120[3]	770[7]	300[7]	11.6[7]<br>0.066[8]	520[7]<br>500[8]		
ADP (+mg2+)	75[7]<br>33[3]	330[7]	130[7]	1.7[7]			
ADP	4[7]<br>28[3]	257[7]	100[7]	25[7]	4[7]		
Ap5A (+mg2+)					0.015[7]	1[11]	
Ap5A					0.1[7]	390[12]	390[12]
Steady-state (+mg2+)			650[8]<br>263[13]				
AMP-PNP + AMP (+mg2+)						286[13]<br>160[14]	1374[13]<br>440[14]
ADP + ADP (+mg2+)						190[15]	
ADP + ADP						0.05[15]	
ADP + AMP (+mg2+)						2800[15]	
ADP + AMP						0.09[15]	

Supplementary Table 8. Estimated rates and 95% confidence intervals (CIs) for the kinetic model for the ATP-induced endosteric closing of the LID and NMP domain presented in (Figure 3). The rates were retrieved by fitting the kinetic data to a Hidden Markov Model (HMM) based on the four observable states and using different ligand concentration. The confidence intervals are approximated using the asymptotic normality of the maximum likelihood estimator. The bounds were computed using the inverse hessian evaluated at the attained log optimum, and subsequently exponentiated to obtain an interval in linear scale.

**Corresponding reactions**	**Estimate**	**95% CI** Lower bound	Upper bound
M → M*; M1 → M1* ($\cdot$|ATP|$^{-1}$)	2.2E+04	*1.4E+04*	*3.5E+04*
M ← M*; M1 ← M1*	2.5E+04	*1.6E+04*	*4.0E+04*
M → M1; M* → M1*	5.0E-01	*4.8E-02*	*5.1E+00*
M ← M1; M* ← M1*	2.0E+01	*1.6E+00*	*2.4E+02*
M* → M2*	4.2E+03	*3.7E+03*	*4.8E+03*
M* ← M2*	7.9E+02	*7.3E+02*	*8.6E+02*
M2* → M2** ($\cdot$|AMP|$^{-1}$)	1.0E+04	*6.0E+03*	*1.7E+04*
M2* ← M2**	4.7E+02	*4.0E+02*	*5.5E+02*
M2** → M**	3.2E+01	*1.6E+01*	*6.3E+01*
M2** ← M**	2.3E+03	*1.7E+03*	*3.0E+03*
M2** → M1**	3.2E+01	*2.2E+01*	*4.5E+01*
M2** ← M1**	1.7E+03	*1.5E+03*	*2.0E+03*
M2** → M3**	5.3E+02	*4.7E+02*	*5.9E+02*
M2** ← M3**	1.2E+03	*1.1E+03*	*1.2E+03*

Supplementary Table 9. Attained log likelihoods for specifications V1, V2, and V3 for models of both ATP+AMP and ADP binding. The magnitudes of differences are attributable to the large size of the experimental datasets. Predictions made by the different models are provided in Supplementary Table 10 and 12, as well as in Supplementary Figures 18 and 22.

	**Log Likelihood**		
**Ligand**	**Model V1**	**Model V2**	**Model V3**
**ATP+AMP**	-1064262	-1055567	-1052792
**ADP**	-1004224	-1003806	-1003676

Supplementary Table 10: AK_2+ binding behavior in the ClyA-AS nanopore with ATP and ATP + AMP with their individual values obtained in this study compared to the rates obtained from the different models. Errors are given as standard deviation of the mean between independent pores (*N* ≥ 3). All current levels were collected in 400 mM KCl, 15 mM Tris, 2 mM $MgCl_2$, pH 7.5 at room temperature (22°C) applying -90 mV (*trans*).

Concentration ligand (µM) ATP	On rate (s^{-1}) Experimental	On rate (s^{-1}) Model V1	On rate (s^{-1}) Model V2	On rate (s^{-1}) Model V3
10	25.6 ± 7.2	31.96436	42.21194	31.26268
20		62.02038	82.04444	61.14218
50	90.8 ± 38.9	152.57324	193.85023	151.14327
100	218.0 ± 7.9	293.56915	369.29412	292.55801
200	356.2 ± 54.8	539.44902	644.92219	547.44169
500	1031.0 ± 257.0	1120.89039	1203.5084	1182.65102
1000	1883.9 ± 315.2	1706.77827	1737.70511	1908.27891

Concentration ligand (µM) ATP	Off rate (s^{-1}) Experimental	Off rate (s^{-1}) Model V1	Off rate (s^{-1}) Model V2	Off rate (s^{-1}) Model V3
10	854.3 ± 256.5	555.76538	786.12025	764.81086
20		545.88761	754.82367	760.07013
50	852.5 ± 59.1	552.89306	762.80077	766.02413
100	799.8 ± 57.0	550.89293	760.06285	761.55648
200	721.4 ± 94.9	551.60849	766.06194	763.55952
500	744.5 ± 25.8	552.86595	768.70577	763.72974
1000	693.4 ± 29.6	551.70176	770.13131	760.39578

Concentration ligand (µM) 1 mM ATP + AMP	Off rate (s^{-1}) Experimental	Off rate (s^{-1}) Model V1	Off rate (s^{-1}) Model V2	Off rate (s^{-1}) Model V3
0	825.2 ± 146.9	551.70176	770.13131	760.39578
10	695.8 ± 151.5	528.06677	570.59809	586.19456
50	245.7 ± 20.6	474.58284	280.01847	319.89386
100	177.9 ± 45.1	444.97613	183.41001	220.03107
200	118.9 ± 36.3	421.65804	110.89857	150.39347
500	109.0 ± 1.8	399.48578	61.61589	98.86489
1000	119.6 ± 13.0	391.9359	43.60949	80.11941

Supplementary Table 11. Estimated rates and 95% confidence intervals (CIs) for the kinetic model for the ADP-induced endosteric closing of the LID and NMP domain presented in (Supplementary Figure 21). The rates were retrieved by fitting the kinetic data to a Hidden Markov Model (HMM) based on the four observable states and using different ligand concentration. The confidence intervals are approximated using the asymptotic normality of the maximum likelihood estimator. The bounds were computed using the inverse hessian evaluated at the attained log optimum, and subsequently exponentiated to obtain an interval in linear scale.

**Corresponding reactions**	**Estimate**	**95% CI**<br>Lower bound	Upper bound
M → M*; M1 → M1* ($\cdot\|ADP\|^{-1}$)	5.6E+04	*1.8E+04*	*1.7E+05*
M ← M*; M1 ← M1*	4.2E+03	*1.9E+03*	*9.4E+03*
M → M1; M* → M1*	5.0E-01	*2.9E-01*	*8.7E-01*
M ← M1; M* ← M1*	2.0E+01	*3.0E+00*	*1.3E+02*
M* → M2*	1.0E+03	*7.3E+02*	*1.4E+03*
M* ← M2*	1.0E+03	*7.8E+02*	*1.3E+03*
M2* → M2** ($\cdot\|ADP\|^{-1}$)	3.1E+04	*2.0E+04*	*4.9E+04*
M2* ← M2**	5.0E+02	*3.3E+02*	*7.5E+02*
M2** → M**	5.0E+01	*3.7E+01*	*6.8E+01*
M2** ← M**	1.2E+04	*8.3E+03*	*1.9E+04*
M2** → M1**	5.0E+01	*3.5E+01*	*7.2E+01*
M2** ← M1**	1.0E+04	*7.0E+03*	*1.4E+04*
M2** → M3**	1.8E+02	*1.4E+02*	*2.3E+02*
M2** ← M3**	3.2E+03	*2.0E+03*	*4.9E+03*

Supplementary Table 12. AK_2+ binding behavior in the ClyA-AS nanopore with ADP with their individual values obtained in this study compared to the rates obtained from the different models. Errors are given as standard deviation of the mean between independent pores ($N \geq 3$). All current levels were collected in 400 mM KCl, 15 mM Tris, 2 mM $MgCl_2$, pH 7.5 at room temperature (22°C) applying -90 mV (*trans*).

**Concentration ligand (µM) ADP**	**On rate (s^{-1}) Experimental**	**On rate (s^{-1}) Model V1**	**On rate (s^{-1}) Model V2**	**On rate (s^{-1}) Model V3**
10	32.6 ± 13.3	89.52363	161.81494	118.13011
20	121.2 ± 18.4	186.25179	316.23401	220.79752
50	272.2 ± 30.1	470.90594	699.30327	485.07022
100	762.2 ± 219.2	789.18324	1139.99299	823.65124
200	1272.0 ± 385.7	1035.21256	1791.30071	1367.44035
500	2706.7 ± 223.4	1180.32201	2807.55487	2568.10831
1000	3456.9 ± 650.1	1221.05205	3773.14088	3905.85001

Concentration ligand (μM) ADP	Off rate (s^{-1}) Experimental	Off rate (s^{-1}) Model V1	Off rate (s^{-1}) Model V2	Off rate (s^{-1}) Model V3
10	805.8 ± 136.3	156.53069	332.39745	625.00134
20	420.5 ± 52.5	158.96316	315.7657	470.98335
50	214.8 ± 27.6	166.48589	260.81214	293.52471
100	167.5 ± 49.4	172.46858	203.11697	202.1295
200	101.9 ± 36.5	177.51918	148.16956	148.71376
500	64.3 ± 9.2	180.30542	96.721	110.88129
1000	65.9 ± 22.0	182.39427	73.55647	98.22771

Supplementary Table 13. Approximate mean emissions per group of states with identical LID/NMP arrangements.

States	Approximate mean emission
M0	-180 pA
M1, M1*, M1**	-210 pA
M, M*, M**	-200 pA
M2*, M2**	-225 pA
M3**	-240 pA

Supplementary References

1. Borglund, E., Brolin, S. E. & Ågren, A. Fluorometric Microassays of Adenylate Kinase, an Enzyme Important in Energy Metabolism. *Ups. J. Med. Sci.* **83**, 81–84 (1978).

2. Vaca, G., Sanchez-Corona, J., Olivares, N., Medina, C. & Cantu, J. M. A simple screening procedure for adenylate kinase, hexokinase and glucose-6-phosphate dehydrogenase deficiencies. *Ann. Genet.* **23**, 190–2 (1980).

3. Sheng, X. R., Li, X. & Pan, X. M. An Iso-random Bi Bi Mechanism for Adenylate Kinase. *J. Biol. Chem.* **274**, 22238–22242 (1999).

4. Van Meervelt, V., Soskine, M. & Maglia, G. Detection of two isomeric binding configurations in a protein-aptamer complex with a biological nanopore. *ACS Nano* **8**, 12826–35 (2014).

5. Huang, G. *et al.* PlyAB Nanopores Detect Single Amino Acid Differences in Folded Haemoglobin from Blood**. *Angew. Chemie Int. Ed.* **61**, (2022).

6. Van Meervelt, V. *et al.* Real-Time Conformational Changes and Controlled Orientation of Native Proteins Inside a Protein Nanoreactor. *J. Am. Chem. Soc.* **139**, 18640–18646 (2017).

7. Reinstein, J. *et al.* Fluorescence and NMR investigations on the ligand binding properties of adenylate kinases. *Biochemistry* **29**, 7440–7450 (1990).

8. Yan, H. & Tsai, M. D. Mechanism of adenylate kinase. Demonstration of a functional relationship between aspartate 93 and magnesium by site-directed mutagenesis and proton, phosphorus-31, and magnesium-25 NMR. *Biochemistry* **30**, 5539–5546 (1991).

9. Sinev, M. A., Sineva, E. V., Ittah, V. & Haas, E. Domain Closure in Adenylate Kinase. *Biochemistry* **35**, 6425–6437 (1996).

10. Ådén, J., Weise, C. F., Brännström, K., Olofsson, A. & Wolf-Watz, M. Structural Topology and Activation of an Initial Adenylate Kinase–Substrate Complex. *Biochemistry* **52**, 1055–1061 (2013).

11. Pelz, B., Žoldák, G., Zeller, F., Zacharias, M. & Rief, M. Subnanometre enzyme mechanics probed by single-molecule force spectroscopy. *Nat. Commun.* **7**, 10848 (2016).

12. Henzler-Wildman, K. A. *et al.* Intrinsic motions along an enzymatic reaction trajectory. *Nature* **450**, 838–844 (2007).

13. Wolf-Watz, M. *et al.* Linkage between dynamics and catalysis in a thermophilic-mesophilic enzyme pair. *Nat. Struct. Mol. Biol.* **11**, 945–949 (2004).

14. Hanson, J. A. *et al.* Illuminating the mechanistic roles of enzyme conformational dynamics. *Proc. Natl. Acad. Sci.* **104**, 18055–18060 (2007).

15. Kerns, S. J. *et al.* The energy landscape of adenylate kinase during catalysis. *Nat. Struct. Mol. Biol.* **22**, 124–131 (2015).
